# Deacetylation of FOXP1 by HDAC7 potentiates self-renewal of mesenchymal stem cells

**DOI:** 10.1186/s13287-023-03376-7

**Published:** 2023-07-28

**Authors:** Shifeng Ling, Tienan Chen, Shaojiao Wang, Wei Zhang, Rujiang Zhou, Xuechun Xia, Zhengju Yao, Ying Fan, Song Ning, Jiayin Liu, Lianju Qin, Haley O. Tucker, Niansong Wang, Xizhi Guo

**Affiliations:** 1 Bio-X Institutes, Key Laboratory for the Genetics of Developmental and Neuropsychiatric Disorders, Ministry of Education, Shanghai Jiao Tong University, Shanghai, China; 2 Department of Nephrology, Sixth People’s Hospital, Shanghai Jiao Tong University, Shanghai, 200240 China; 3 State Key Laboratory of Reproductive Medicine, Center of Clinical Reproductive Medicine, The First Affiliated Hospital of Nanjing Medical University, Jiangsu Province Hospital, Nanjing, China; 4 Institute for Cellular and Molecular Biology, University of Texas at Austin, 1 University Station A5000, Austin, TX 78712 USA

**Keywords:** Mesenchymal stem cells, Self-renewal, Deacetylation, FOXP1, HDAC7

## Abstract

**Background:**

Mesenchymal stem cells (MSCs) are widely used in a variety of tissue regeneration and clinical trials due to their multiple differentiation potency. However, it remains challenging to maintain their replicative capability during in vitro passaging while preventing their premature cellular senescence. Forkhead Box P1 (FOXP1), a FOX family transcription factor, has been revealed to regulate MSC cell fate commitment and self-renewal capacity in our previous study.

**Methods:**

Mass spectra analysis was performed to identify acetylation sites in FOXP1 protein. Single and double knockout mice of FOXP1 and HDAC7 were generated and analyzed with bone marrow MSCs properties. Gene engineering in human embryonic stem cell (hESC)-derived MSCs was obtained to evaluate the impact of FOXP1 key modification on MSC self-renewal potency.

**Results:**

FOXP1 is deacetylated and potentiated by histone deacetylase 7 (HDAC7) in MSCs. FOXP1 and HDAC7 cooperatively sustain bone marrow MSC self-renewal potency while attenuating their cellular senescence. A mutation within human FOXP1 at acetylation site (T176G) homologous to murine FOXP1 T172G profoundly augmented MSC expansion capacity during early passages.

**Conclusion:**

These findings reveal a heretofore unanticipated mechanism by which deacetylation of FOXP1 potentiates self-renewal of MSC and protects them from cellular senescence. Acetylation of FOXP1 residue T172 as a critical modification underlying MSC proliferative capacity. We suggest that in vivo gene editing of FOXP1 may provide a novel avenue for manipulating MSC capability during large-scale expansion in clinical trials.

**Supplementary Information:**

The online version contains supplementary material available at 10.1186/s13287-023-03376-7.

## Introduction

Bone marrow mesenchymal stem cells (BM MSCs) constitute a specific stem cell population that accounts for bone regeneration and homeostasis. As initially characterized by Friedenstein (1974), BM MSCs have the capacity of self-renewal and tri-lineage differentiation that can give rise to osteoblasts, chondrocytes, and adipocytes. During development, MSCs, preferentially designated as skeletal stem cells (SSCs), were identified in growth plates and within the periosteum of developing bone [1–5]. MSCs also are the primary stem cells within the perivascular vicinity of adult BM—a tissue that can be specifically targeted by *Prx1-Cre* [6]. In addition, MSCs possess stromal immunomodulatory capacities that allow them to inhibit proliferation and function of several major categories of hematopoietic cells; thus, providing cell sources for tissue regeneration and immune therapies [7–9].

These in vitro self-renewal and expansion properties of MSCs conversely correlate with cellular senescence—a state of durable and irreversible growth arrest [10]. Alterations of oxidative stress and epigenetic modifications, which also drive cellular senescence in stem cells, are partially controlled by SIRT1/FOXO transcriptional pathways [11, 12]. For example, inhibition of SIRT1, a Class III histone deacetylase (HDAC), deteriorates the self-renewal and differentiation capacities of BM MSCs [13–15]. Several additional SIRT family proteins have similar functions in protecting MSCs from senescence. SIRT6 safeguards human MSCs from oxidative stress-induced senescence by coactivating NRF2, an essential transcription factor that regulates an array of detoxifying and antioxidant defense genes in the liver [16]. SIRT3, on the other hand, enhances MSC longevity and differentiation capacity against oxidative stress [17–19], whereas SIRT7 acts to protect human stem cells from aging by stabilizing their heterochromatin [20].

SIRT1 deacetylates Forkhead Box O3 (FOXO3), a crucial transcriptional effector of insulin/insulin-like growth factor (IGF)/SIRT signaling with anti-aging potency [21, 22], in response to oxidative stress [11]. FOXO family proteins also are deacetylated and activated by Class IIa HDACs (HDAC4, 5 and 7) in regulating glucose homeostasis [23]. Therefore, tipping FOXO-dependent responses away from apoptosis and toward stress resistance is a promising strategy for promoting MSC self-renewal and for increasing organism longevity. For instance, phosphorylated sites engineered FOXO3 into human MSCs enhance their stress resistance, attenuate cellular senescence, and promote cardiac repair after myocardial infarction [24, 25].

FOXO-induced apoptosis is regulated via a negative feedback loop by Forkhead Box P1 (FOXP1) [26]. The FOXP1 transcription factor also controls self-renewal potency and quiescence in multiple stem cell lineages, including embryonic stem cells [27], hematopoietic stem cells [28, 29], hair follicle and mammary stem cells [30, 31], neural stem cells [32] and lung epithelial stem cells [33]. Our previous studies revealed an age-dependent role for FOXP1 in maintaining MSC self-renewal potency through direct transcriptional repression of *p16*, a tumor suppressor which plays an important role in cell cycle regulation [34].

In contrast to the extensive body of study directed toward FOXP1 transcription and function (recently reviewed by [35]), its protein modification and the effects thereof are largely unknown. In this report, we show that FOXP1 is deacetylated and stabilized by HDAC7 to promote self-renewal of BM MSCs. A single deacetylation event at FOXP1 residue T172 site within its glutamine-rich domain (amino acids 140–261) is key in manipulating MSC replicative capacity. Our findings reveal a potential site for FOXP1 protein which could be engineered in vivo to increase MSCs expansion capacity.

## Materials and methods

### Mouse models

*Foxp1*^*fl/fl*^ [36] and *Prx1-Cre* [37] mice were generated as described elsewhere. The genetic backgrounds of all mice were C57BL/6N. Mice were bred in pathogen-free conditions with standard rodent chow food (Nanjing Xietong, # 1,010,009). Male mice were used in the experiments unless otherwise indicated. The experiments were not randomized, and the investigators were not blinded to allocation during experiments or outcome assessments. The housing temperature of cages was 20 ~ 22 °C, coupled with 12-h light (07:00–19:00 h) and 12-h dark cycles (19:00–07:00 h). Mice were anesthetized unconsciously by CO_2_, captured with image by cameras (Canon, DS126231), and decapped before harvesting of tissues. All animal experiments were performed according to the guidelines (SYXK 2011–0112) and received ethical approval from committee of Bio-X Institutes of Shanghai Jiao Tong University.

All the reporting of animal experiments adheres to the ARRIVE guidelines.

### Plasmids and retroviral packaging

For expression plasmids, cDNA of FOXP1 and FOXP1^T172G^ was cloned into the pcDNA3.0-His vector, and HDAC7 cDNA was cloned into the pcDNA3.0-Flag vector. For retroviral plasmids, FOXP1 and HDAC7 cDNA were cloned into pMSCV-puro vector. To package retrovirus, pMSCV-FOXP1-puro, pMSCV-HDAC7-puro, and pMSCV-GFP-puro were co-transfected with Gag-Pol and VSV-G into HEK293T cells using Lipo 8000 (Beyotime). After 48 h, culture supernatants were collected and used for hMSCs infection. Stable transformants of hMSCs were selected with 2 μg/mL puromycin. Then, western blot and qPCR were used to detect the expression of FOXP1 and HDAC7.

### Generation of HDAC7.^±^ mice by CRISPR/Cas9

A 142 bp-fragment was deleted in *HDAC7* exon 2 downstream of ATG, resulting premature stop of protein translation. A chemically modifying sgRNA (TGCCCTCCAGCCAGACACACC) (Genscript) for HDAC7 was synthesized, and spCas9 protein was purchased from NovoProtein (# E365-01A). The generation of *HDAC7*-deficient mice by CRISPR/Cas9 was performed as a protocol previously described [38]. Briefly, the zygotes of mouse embryos were isolated from the oviduct at 0.5 d post-coitum (dpc) and washed clean of cumulus cells. The 50 μl RNP mixture of sgRNA (25-50 ng/μl) and spCas9 protein (50-100 ng/μl) was introduced into mouse zygotes by microinjection. Founder mice (C57BL/6N X ICR) were genotyped by PCR amplification and crossed with C57BL/6N mice to generate N_1_ germline mice. The N_1_ mice were genotyped by PCR and backcrossed with C57BL/6N mice for at least 4 generations prior to strain expansion. The genotyping primers of all mice are listed in Additional file 1: Table s1.

### Human ESC and MSC cells and cell culture

Mouse MSCs were extracted from bone marrow and expanded using a mouse MesenCult Proliferation Kit (STEMCELL Technologies) according to the manufacturer’s protocols. Briefly, mouse bone marrow cells from tibias and femurs were flushed out with 2 ml DMEM plus 10% FBS. Nucleated cells were counted using methylene blue dissolved in 3% acetic acid (STEMCELL Technologies). Cell lines 293 T and C3H10T1/2 (obtained from ATCC) were cultured in DMEM with 10% FBS. Diffuse Large B-Cell Lymphomas (DLBCLs) were the kind gift of Dr. Louis Staudt (Center for Cancer Research, National Cancer Institute, Bethesda MD. USA). hMSCs (UMSC 10.2.2) were established from human amnion and contributed by Prof. Lianju Qin in the First Affiliated Hospital of Nanjing Medical University. The stem cell identity, genetic safety, biological safety, toxicology, tumorigenicity, pluripotency, biological activity, and safe dose were completely assessed and all tested items were up to standard. The hMSCs were cultured with α-MEM plus 10% FBS and 1% Glutamine during lentiviral transfection.

A human ESC cell line CCRM-hESC-22 was generated as described previously [39] and contributed by Prof. Lianju Qin in the First Affiliated Hospital of Nanjing Medical University. For gene editing in this study, hESCs were cultured with PGM1 medium (Cellapy) as previously described [40].

The detailed procedures of generation of *FOXP1*^*T176G*^ hESCs and directed differentiation into hMSCs are described in Additional file 1: Materials and Methods.

## Evaluation of tri-lineage differentiation of MSCs, and generation of ***FOXP1***^T176G^ hESCs

Cells were differentiated toward osteoblasts, chondrocytes, and adipocytes as indicated in Additional file 1: Materials and Methods.

### μCT analysis

Femurs were dissected from mice and kept in 70% ethanol at 4 °C, prior to fixation in 4% PFA for 24 h. μCT scanning of bone was performed on a SkyScan 1176 (Bruker). A 3D model was reconstructed, and structural indices were calculated using CTAn software. ROIs (regions of interest) were selected 5 mm below bone growth plates.

### Co-IP

For in vitro coimmunoprecipitation (Co-IP), His, Flag, or Myc-tagged proteins were produced in 293 T cells transfected by Lipo 8000 with the corresponding plasmids. For in vitro co-IP, His- or FLAG-tagged proteins were produced in HEK293T cells transfected with corresponding plasmids. For in vivo co-IP, BM MSCs or DLBCLS were isolated and cultured from adult wild-type mice or from established lines obtained from human donors. Total cell lysates were incubated overnight at 4 °C with antibodies (described below) or normal IgG (Santa Cruz Biotechnology Inc., sc-2027) as control. Protein complexes were precleared with Protein A/G PLUS-Agarose (Santa Cruz Biotechnology Inc., sc-2003), washed several times, then boiled and analyzed by western blotting.

### Mass spectra analysis

For mass spectrum analysis, FOXP1 was over-expressed in HEK293T cells; 48 h later, the cells were treated with NP40 lysis buffer. Then, total cell lysates were incubated overnight at 4 °C with anti-His. Protein complexes were precleared with Protein A/G PLUS-Agarose (Santa Cruz Biotechnology Inc., sc-2003), washed several times and boiled. Proteins were fractionated on SDS-PAGE and then Western blotted with anti-FOXP1 antisera. The peptides then were analyzed with an EASY-nL 1200 system coupled with a Q Exactive plus mass spectrometer (Thermo Scientific, Bremen, Germany), as described in supplemental information with details. The raw MS data of FOXP1 protein modifications were deposited in PeptideAtlas (dataset Identifier: PASS03792). The detailed procedures of mass spectra analysis are described in Additional file 1: Materials and Methods.

### Western blotting

Cells were lysed by T-PER lysis buffer (Thermo Scientific) for 15 min in 4℃. Protein samples were separated by SDS-PAGE, transferred to Immobilon-PVDF membrane (Millipore), blocked by 5% non-fat milk (Sangon), and incubated with primary antibodies against FOXP1 (Millipore, ABE68, 1:1000), His-tag (Transgen, HT501-01, 1:1000), Flag (Earth OX, E022060-02, 1:1000), HDAC7 (ABclonal, A13008, 1:1000), Acetyl lysine (PTM Bio, PTM-101, 1:1000), GFP (Transgen, HT801-01, 1:1000), Actin (Transgen, HC201-01, 1:1000) at 4℃ overnight. Proteins were visualized using horseradish peroxidase–conjugated (HRP-conjugated) secondary antibody and chemiluminescent HRP substrate (Share-bio).

### Immunofluorescence

Cultured MSCs or 293 T cell lines were fixed in 4% PFA for 15 min at room temperature (RT). Cells were blocked with 5% normal goat serum at RT for 1 h, cells were then incubated with antibodies against γ-H2AX(Gene Tex, GTX11174, 1:200), LAP2 (BD, 611,000, 1:200), His (Transgen, HT501-01, 1:200), Flag (Earth OX, E022060-02, 1:1000), followed by the incubation with Alexa Fluor 488- or Alexa Fluor 594-conjugated secondary antibodies. The white field or fluorescence images were acquired with resolution more than 800dpi through microscopes DMI3000B (Leica), ECLIPSE 80i (Nikon) and Microsystems CMS GmhH (Leica). Images analyzed by Adobe Photoshop 2020 with average resolution more than 500dpi.

### Luciferase reporter assay

Luciferase assays were performed in C3H10T1/2 cell lines. The *p16*-*Luc* and *RBPjκ-Luc* reporter plasmids have been described [34]. Luciferase and expression plasmids of FOXP1 or HDAC7 were co-transfected into C3H10T1/2 cell lines. Cells were transfected using Lipo 8000 (Beyotime) in 24-well plates. The transfection amount of each plasmid was 200 μg, and the total amount of plasmid DNA was balanced among each transfection with pcDNA3.0 vector where necessary. After 48 h, dual luciferase assay was performed according to the manufacturer’s protocols (Vazyme).

### Senescence-associated beta-galactosidase (SA-βgal) staining

SA-β-gal staining assay was performed according to the manufacturer’s protocols (Beyotime). Briefly, cells were fixed for 10 min at RT and then stained overnight at 37℃. The percentages of SA-β-gal positive cells were quantified microscopically.

### qPCR

Total RNA was extracted with Trizol (Vazyme). Reverse transcriptase was used for cDNA generation by employing the Hiscript III reverse transcription system (Vazyme). RT-qPCR was performed on a real-time PCR system (Roche 480) using SYBR Green (Vazyme). The primer sequences are listed in Additional file 1: Table s1.

### Statistical analysis

All data are presented as mean ± SEM. Two-tailed Student's t tests were employed for comparisons between two groups, and two-way ANOVA was used for multiple group comparisons. *, *P* ≤ 0.05; **, *P* ≤ 0.01; ***, *P* ≤ 0.001; ns, not significant.

## Results

### Multiple acetylation and deacetylation of FOXP1 by HDAC7

**Fig. 1:**
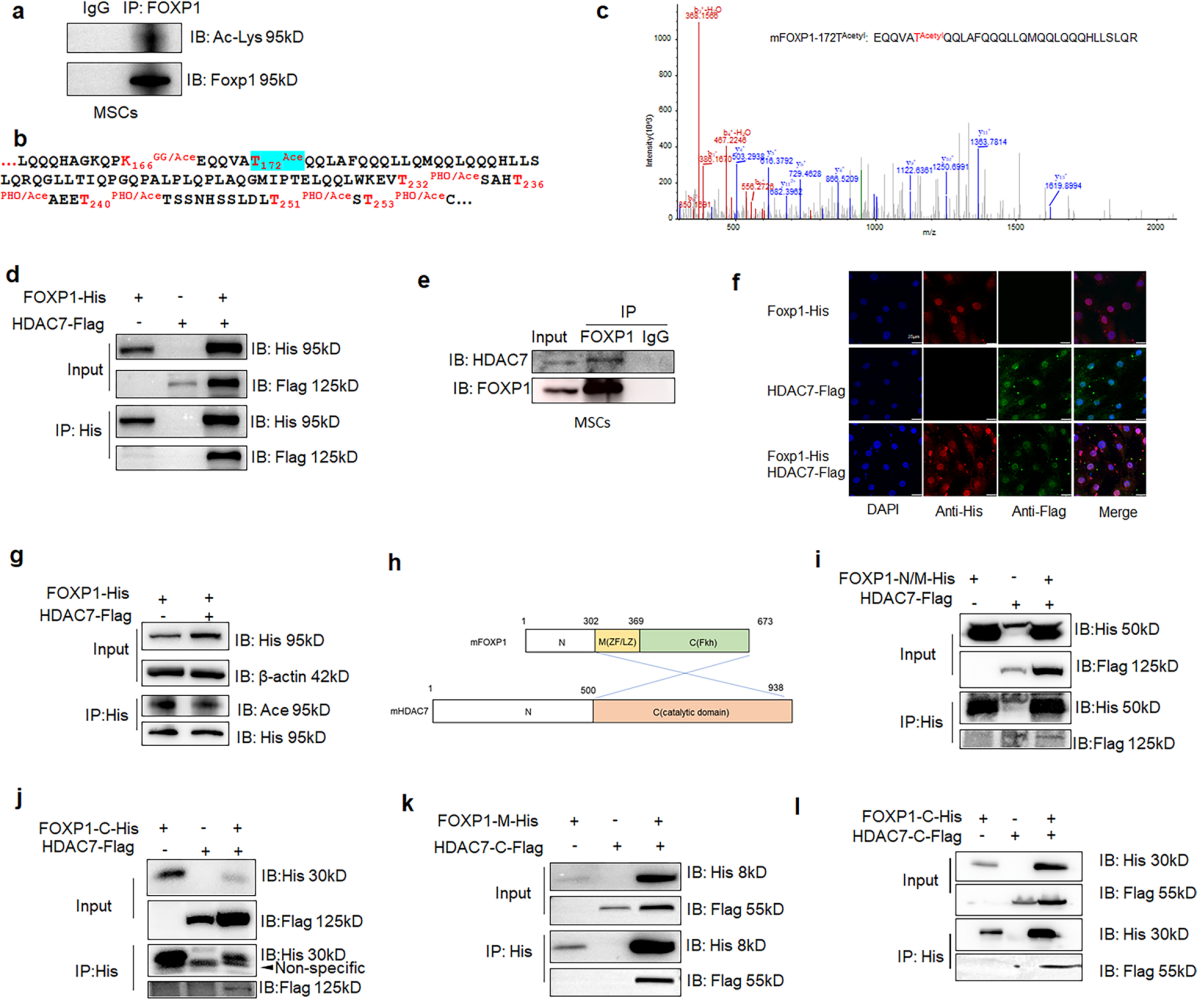
Deacetylation of FOXP1 by HDAC7. **a** FOXP1 protein was enriched by immunoprecipitation (IP) from mesenchymal cells of human mesenchymal stem cells (MSCs) and was detected for acetylation by western blot with anti-Pan Acetyl-Lysine antibody (Ab). **b** A pcDNA-FOXP1-His plasmid was expressed in 293 T cells and FOXP1 was harvested via IP with anti-His Ab. Extensive acetylation was identified by mass spectrum analysis. **c** Mass spectral characterization of murine FOXP1 protein acetylation at amino acid T172. **d** Co-IP detection of the in vitro interaction of FOXP1 and HDAC7 in 293 T cells following their transfection with the indicated plasmids (representative of 3 independent experiments). **e** Co-IP detection of the in vivo interaction of FOXP1 and HDAC7 in bone marrow (BM) MSCs (representative of 3 independent experiments). **f** Immunostaining with anti-His (green) and anti-Flag (red) detects the co-localization of FOXP1 and HDAC7 within perinuclear region of C3H10T1/2 cells transfected with the indicated plasmids. Bar, 25 μm. **g** Deacetylation of FOXP1 by HDAC7 in 293 T cells. **h** Diagram depicting the expression subregions of FOXP1 protein. FOXP1-N: FOXP1 protein N-terminal(1-302aa); FOXP1-M: middle part (302-369aa), including zinc finger (ZF) and leucine zipper (LZ) domains; FOXP1-N/M: N-terminal and Middle part (1-369aa); FOXP1-C: C-terminal (369-673aa), containing the Forkhead (Fkh) domain [FOXP1-C(Fkh)]. Blue lines indicated the potential interaction domain between FOXP1 and HDAC7 protein. **i–l** Co-IP detection of the in vitro interaction of FOXP1-N/M and HDAC7-C in 293 T cells transfected with the indicated plasmids (representative of 3 independent experiments)

At least four FOXP1 isoforms have been identified in various vertebrate tissues. To investigate their potential protein acetylation, one of these (isoform 2; 673aa; GenBank: AAH64764.1) was pulled down in primary human mesenchymal stem cells (hMSCs) by immunoprecipitation (IP) with anti-FOXP1 antibody. We observed that FOXP1 protein was extensively acetylated as detected with an anti-Pan Acetyl-Lysine antibody (Fig. 1a). Next, His-tagged, full-length FOXP1 protein was harvested from 293 T cells following transfection with a pcDNA-FOXP1-His plasmid. Mass spectrum analysis revealed numerous acetylation sites within key FOXP1 domains, including its glutamine-rich domain (QR, amino acids 140–261), its zinc finger (ZF, residues 302–327), its leucine zipper (LZ, 341–369) and its Foxhead DNA binding domain (Fkh, 461–555). These domains are highly conserved in FOXP (1–4) family proteins (Additional file 1: Fig. S1a). Singly acetylated residues include K10, T172, T273, R370, T386, T404, T423, R434, R461, K517, and T531 (Fig. 1b, c; Additional file 1: Fig. S1b). Of note, several sites were detected with dual modification of phosphorylation/acetylation or ubiquitination/acetylation, including K166, T232, T236, T240, T250, T253, K305, K438 and K480.

The 18 human Histone Deacetylases (HDACs) are divided into shared homology groups, including Class I (HDAC1-3 and 8), Class II (HDAC4, 5–7, 9 and 10), Class III (SIRT1-7), and Class IV (HDAC11) [41]. We cloned the cDNA encoding each of these HDACs into pcDNA expression vectors tagged with either FLAG- or His-tags at their C-termini. To identify which of these HDACs are specific modifiers of FOXP1, Co-IP was performed in 293 T cells co-transfected with pcDNA-FOXP1-His and pcDNA-HDAC-FLAG expression plasmids. As shown in Fig. 1d and e, only HDAC7 interacted specifically with FOXP1, both in vitro and in vivo. Immunostaining with anti-FLAG or anti-His Abs in C3H10T1/2 cells co-transfected with FOXP1/HDAC7 expression plasmids identified the interaction within the perinuclear region (Fig. 1f). HDAC7 association also decreased the acetylation level of FOXP1 in vitro, as evidenced by western blot with anti-Pan Acetyl-Lysine antibody in 293 T cells (Fig. 1g), as well as by MS analysis (Additional file 1: Fig. S1c).

To assist in identifying more precisely the HDAC7 site, we divided murine FOXP1 into three sections: N-terminal (N; residues 1–301 which span the QRF region), the middle residues (M, residues 302–369) which include the zinc finger plus leucine zipper and the C-terminal residues (Fkh, residues 370–673) which span the Forkhead domain (Fig. 1h). We also divided murine HDAC7 into N-terminal (residues1-500) and the C-terminal catalytic domain (residues 501–938). Co-IP performed between each of the truncated domains revealed the strongest FOXP1-HDAC7 interaction to be within the zinc finger/leucine zipper of FOXP1 and the C-terminus of HDAC7 (Fig. 1i–l).

Collectively these findings indicate that FOXP1 interacts with and is specifically deacetylated by HDAC7 in mesenchymal cells.

### HDAC7 stabilizes FOXP1 from ubiquitination-mediated degradation

**Fig. 2:**
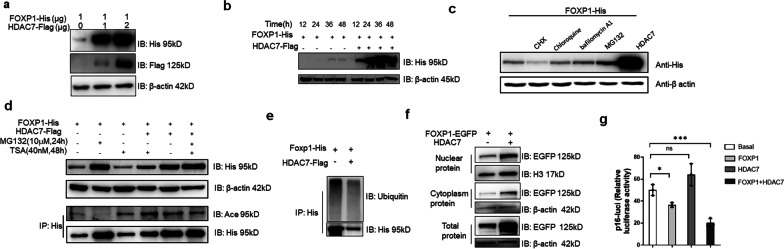
HDAC7 facilitates FOXP1 protein stabilization. **a** The expression of FOXP1 protein increased as the dosage of HDAC7 increased. Different levels (0, 1 μg and 2 μg) of HDAC7 and FOXP1 (1 μg) expression plasmids were transfected into 293 T cells and then analyzed 24 h later. β-actin levels served as loading control here and in subsequent sub-figures. **b** The expression of FOXP1 increased following harvesting from 293 T cells post-transfection at the indicated times (12, 24, 36, 48 h) with the indicated plasmids (2 μg). **c** FOXP1 expression assessed in 293 T cells that were treated for 12 h with either Cycloheximide (CHX, an inhibitor of protein synthesis, 10 μg/ml), or Chloroquine (an inhibitor of lysosomal activity, 50 μM), or Bafilomycin A1 (an inhibitor of autophagy, 100 μM) and with MG132 (a proteasome inhibitor, 10 μM). **d** Stabilization of FOXP1 by HDAC7 is sensitive to HDAC inhibitor TSA. 293 T cells transfected with the indicated plasmids were treated with 40 nM TSA for 48 h, or 10 μM MG132 for 24 h prior harvesting of FOXP1 via its His tag. FOXP1 acetylation was assessed simultaneously (as described in the legend for Fig. 1). **e** HDAC7 reduces FOXP1 ubiquitination levels in 293 T cells transfected with the plasmids indicated on the figure. **f** HDAC7 facilitates FOXP1 accumulation both within the nucleus and the cytoplasm of 293 T cells transfected with the indicated plasmids. **g** Luciferase reporter assays demonstrate that FOXP1 transcriptional repression of *p16* is enhanced by HDAC7. C3H/10T1/2 cells were co-transfected with *p16-Luc*, FOXP1-His, and/or HDAC7-Flag. Reporter activity is presented as relative luciferase units (RLU). Data shown are representative of 3 independent experiments). *, *P* ≤ 0.05; **, *P* ≤ 0.01; ***, *P* ≤ 0.001; ns, non-significant

To determine the functional consequence of HDAC7 modification and interaction with-FOXP1, we varied the expression levels of HDAC7 in transfected 293 T cells by increasing the input concentration of pcDNA-HDAC7-FLAG plasmid from 0 to 5 μg (Fig. 2a). In addition, we measured FOXP1 expression levels over a post-transfection time course of 12–48 h (Fig. 2b) using inputs of 2ug of pcDNA-FOXP1-His and pcDNA-HDAC7-FLAG. As shown in Fig. 2a and b, FOXP1 protein levels increased in the presence of HDAC7 in dose- and time-dependent manners.

Next 293 T cell cultures transfected with and expressing tagged FOXP1 were treated for 24 h with cycloheximide (CHX, an established inhibitor of protein synthesis), or with Chloroquine (an inhibitor of lysosomal activity), or with Bafilomycin A1 (an inhibitor of autophagy) or with MG132 (a proteasome inhibitor). As shown in Fig. 2c, only MG132 treatment was capable of increasing FOXP1 protein levels. FOXP1 levels were stabilized following 24-h exposure to MG132 (10 μM) to a similar extent as that of transfection with HDAC7 (Fig. 2d; compare lanes 2 and 5). Yet the function of HDAC7 in stabilization of FOXP1 could be reversed by a 48-h exposure to 40 nM trichostatin A (TSA), an inhibitor of HDAC (Fig. 2d, Lanes 4 and 5). In addition, the presence of HDAC7 markedly diminished the gross ubiquitination level of FOXP1, and in particular, the polyubiquitination modifications (Fig. 2e). Meanwhile, exposure to HDAC7 enhanced FOXP1 accumulation in both the nucleus and the cytoplasm (Fig. 2f). The classical cellular senescence marker, *p16*, was previously reported to be a direct readout of FOXP1 transactivation activity in MSCs [34]. A luciferase reporter assay (detailed in Materials and Methods) confirmed that HDAC7 also enhanced the repressive activity of FOXP1 (Fig. 2g).

Taken together, these data demonstrate that HDAC7 facilitates FOXP1 stabilization and activates its function, possibly by protecting it from proteasome-dependent degradation.

### Deletion of FOXP1/HDAC7 in MSCs exacerbates bone loss

FOXP1 controls cell differentiation and self-renewal of MSCs in an age-dependent manner [34]. To test the effect of HDAC7 on FOXP1 in MSC self-renewal and differentiation, we inactivated both of them in BM MSCs by crossing a floxed (*fl)* allele, *Foxp1*^*fl/fl*^, with *Prx1-Cre* and *HDAC7*^+/−^ C57BL/6N mice. The conditional *Fox1*^*fl/fl*^ allele was floxed within Foxhead domain [36]. The *Prx1-Cre* transgenic mice could express Cre activity specially targeted MSCs within perivascular region of bone marrow [37]. *Prx1-Cre;Foxp1*^*fl/fl*^ conditional Knockout (cKO) mice were hereafter designated as *Foxp1*_*Prx1*_^Δ*/*Δ^.

*HDAC7*^+/−^ mice were generated by CRISPR/Cas9, which was primed by a sgRNA aligning just upstream of the translation start site at Exon 2 of *HDAC7* (Additional file 1: Fig. S2a). In *HDAC7*^+/−^ mice, a genomic fragment containing the ATG codon in Exon 2 was deleted, leading to an out-of-frame codon and to premature termination of HDAC7 translation, which mimic the strategy as previously described [42]. The knockout efficiency was evidenced by extensive down-regulation of HDAC7 mRNA and protein (Additional file 1: Fig. S2b, c).

**Fig. 3:**
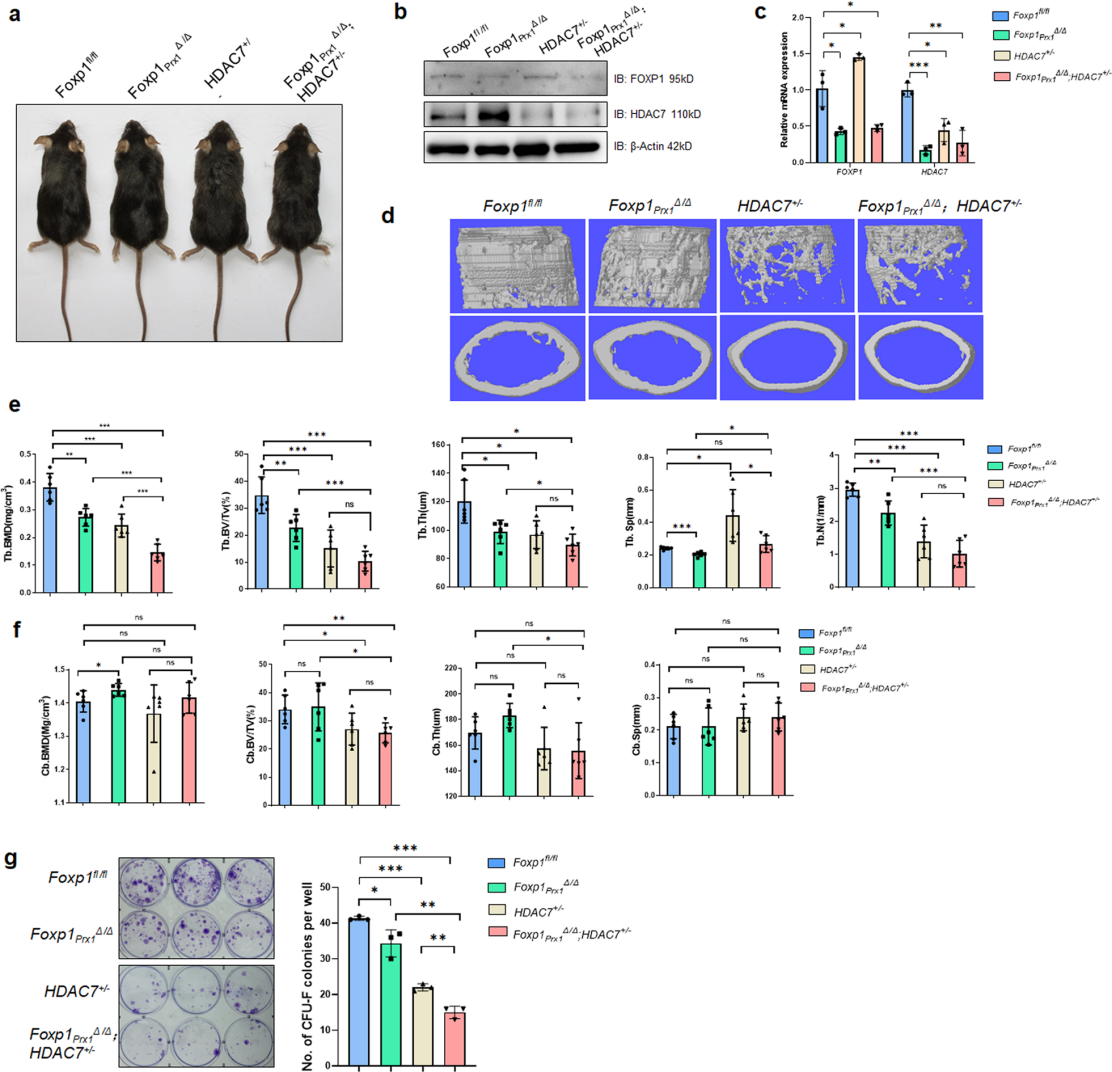
Knockout of *FOXP1/HDAC7* exacerbates bone mass loss. **a** Representative dorsal view of 3-month-old *Foxp1*^*fl/fl*^, *Foxp1*_*Prx1*_^*∆/∆*^, *HDAC7*^+/−^and *Foxp1*_*Prx1*_^*∆/∆*^*;HDAC7*^+/−^ double KO mice. **b** Western blot detection of FOXP1 and HDAC7 within BM MSCs of mice at 3 months of age. **c** qPCR validation of mRNA level of FOXP1 and HDAC7 in MSCs from mice at 3 months of age. Data shown are representative of 3 independent assays. **d** 3D view of cortical and trabecular bone structure by µCT analysis of femur bones of mice at 3 months of age. **e–f** Quantification of cortical and trabecular bone properties by µCT analysis (n = 6). Abbreviations: BV/TV, bone volume/tissue volume; BMD, bone mineral density; Tb. N., trabecular bone number; Tb. Sp., trabecular bone spacing; Tb. Th., trabecular bone thickness; BS, bone surface; Cb. Th., cortical bone thickness. Two-tailed Student's t tests for comparisons between two groups. Each group consists of 9 mice of each phenotype. **g** Crystal purple staining of CFU-F colonies of MSCs from 3-month-old mice (n = 3) of the indicated genotypes.*, *P* ≤ 0.05; **, *P* ≤ 0.01; ***, *P* ≤ 0.001; ns, non-significant

These single and double KO mice showed no variation in size, weight nor growth as compared to *Foxp1*^*fl/fl*^ controls (Fig. 3a, Additional file 1: Fig. S2d and data not shown). qPCR and western blot analyses confirmed efficient depletion of *Foxp1* and *HDAC7* expression (Fig. 3b and c).

Next, we analyzed each single and double cKO mouse for both trabecular and cortical bone parameters using microcomputer tomography (μCT). As shown in Fig. 3d and e, a significant loss of trabecular bone mass was detected in both *Foxp1*_*Prx1*_^Δ*/*Δ^ and *HDAC7*^+/−^ mice at 3 months of age, as evidenced by a decline in trabecular bone mineral density (Tb. BMD), bone volume/total volume (Tb. BV/TV), bone thickness (Tb. Th) and trabecular bone number (Tb. N) as well as by a modest increase in trabecular bone space (Tb. sp). Of note, the trabecular bone loss was exacerbated for each of these parameters in double cKO (*Foxp1*_*Prx1*_^Δ*/*Δ^*; HDAC7*^±^) mice. By contrast, no evident defect was observed in these properties within cortical bones of single and double cKO mice (Fig. 3f).

These findings indicate that FOXP1 and HDAC7 provide an additive role in bone mass maintenance.

### FOXP1/HDAC7 deficiency perturbs differentiation and self-renewal potency of MSCs

Bone mass accrual primarily stems from MSC expansion and osteogenic differentiation. Therefore, we first examined the differentiation capacity of MSCs, flushed from BM of single and double cKO mice and induced to differentiate in vitro. Within 14 to 21 days post-osteogenic induction, alkaline phosphate (ALP) and alizarin red (ARS) staining showed a relative decrease in osteogenic differentiation of MSCs from *Foxp1*_*Prx1*_^Δ*/*Δ^ mice (Additional file 1: Fig. S3a and b, left panels). These results were in line with what we observed previously [34]. HDAC7 was previously shown to act as a co-repressor of Runx2 during osteoblast differentiation [43]. Parallel with those findings, we observed a marked augmentation of the osteogenic capacity of MSCs from *HDAC7*^+/−^ and double cKO mice as compared to controls (Additional file 1: Fig. S3a and b). This change in MSC differentiation potency was validated by parallel alterations of osteoblast-specific *Runx2* transcripts as well as those of *Alp*, *Col1a1 and Osterix*. Of note, *HDAC7* deficiency appeared to have no significant effect on osteoblast maturation, as revealed by only modest alterations in expression levels of the typical osteocyte markers, *SOST* and *DMP1* (Additional file 1: Fig. S3a and b, right panels). The adipogenic potency of MSCs also was increased in *HDAC7*^+/−^ and double KO mice as compared to controls (Additional file 1: Fig. S3c). But their chondrogenic potency was barely altered (Additional file 1: Fig. S3d).

We conclude from these experiments that deficiency of both *FOXP1* and *HDAC7* enhances early osteogenic differentiation of MSCs, but have little effect upon osteoblast maturation and osteocyte formation.

**Fig. 4:**
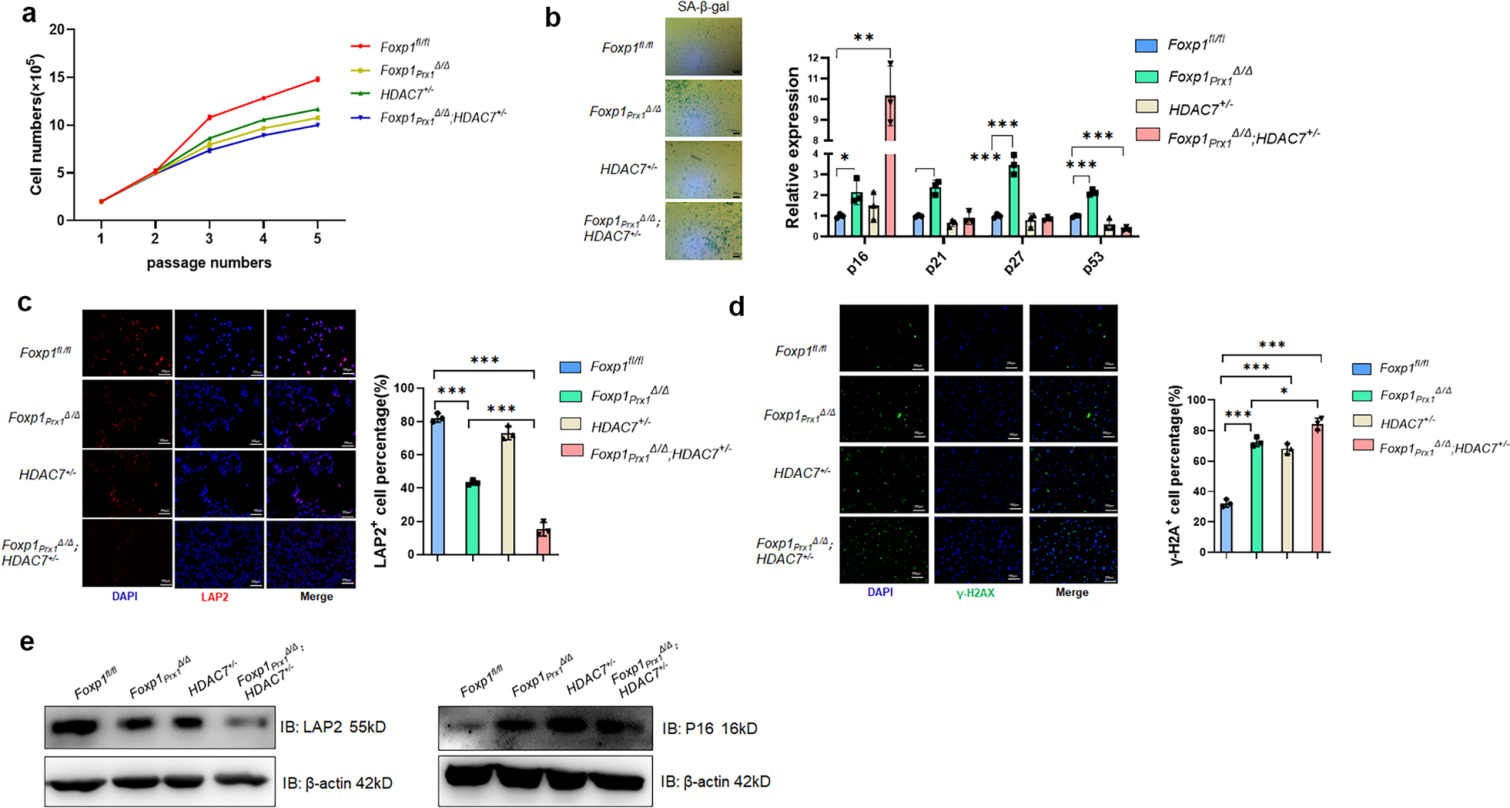
*FOXP1/HDAC7* deficiency impairs the self-renewal potency of MSCs **a** Population doubling curve of BM MSCs of *Foxp1*^*fl/fl*^, *Foxp1*_*Prx1*_^*∆/∆*^, *HDAC7*^+/−^, and *Foxp1*_*Prx1*_^*∆/∆*^*;HDAC7*^+/−^ 3-month-old transgenic mice. Each group consists of 3 mice of each phenotype. **b** SA-β-gal staining (left panel) and qPCR analysis (right panel) for cellular senescence markers at passage 5 of MSCs in transgenic mice of each genotypes (n = 3) indicated in panel A. Bar, 200 μm. **c** Immunofluorescence of LAP2 (red) and DAPI (blue) for passage 5 MSCs from transgenic mice (n = 3) of genotype indicated in (a). Right panel shows the quantification of LAP2-positive cells. Bar, 100 μm. **d** Immunofluorescence of γH2AX (Green) and DAPI (blue) for passage 5 MSCs from knockout mice (n = 3) of each genotype indicated in (a). Right panel shows the quantification of γH2AX-positive cells. Bar, 100 μm. *, *P* ≤ 0.05; **, *P* ≤ 0.01; ***, *P* ≤ 0.001; ns, non-significant. **e** Detection of LAP2 and P16 protein expression in passage-5 MSCs from bone marrows of *Foxp1*^*fl/fl*^, *Foxp1*_*Prx1*_^*Δ/Δ*^, *HDAC7*^+/−^, and *Foxp1*_*Prx1*_^*Δ/Δ*^*;HDAC7*^+/−^ mice (n = 3)

Most importantly, however, the self-renewal capacity of MSCs was additively impaired upon loss of FOXP1 and HDAC7, as evidenced by loss of colony-forming unit fibroblasts (CFU-F) (Fig. 3g). In agreement with these data, in vitro expansion of MSCs was arrested within each single or double cKO mice compared to controls as measured by their rates of population doubling (Fig. 4a).

**Fig. 5:**
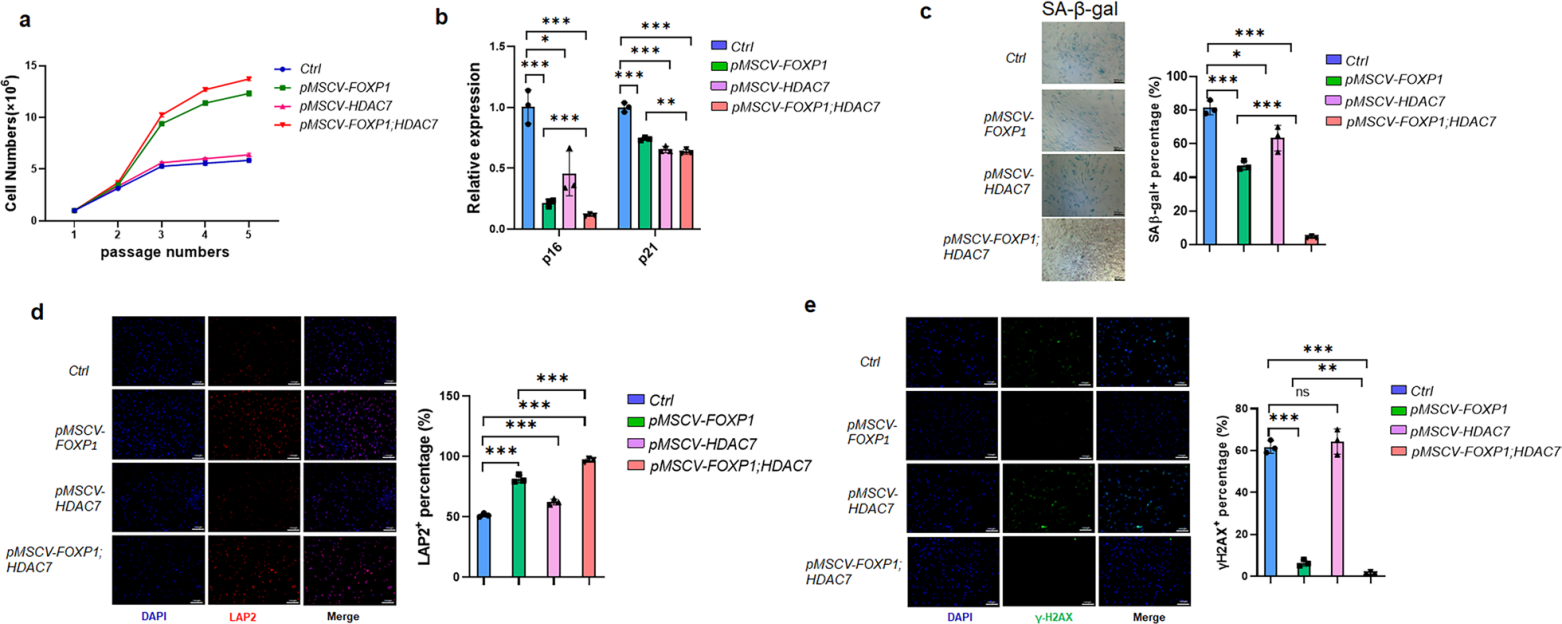
*FOXP1/HDAC7* retroviral-mediated overexpression enhances the expansive potency of hMSCs. **a** Population doubling curves of hMSCs infected with retroviral *pMSCV-FOXP1* and/or *pMSCV-HDAC7.* Human (h)MSCs were infected with *pMSCV* empty vector as a control. Data shown are representative of 3 independent replications. **b** qPCR analysis of *p16/p21* expression in hMSCs overexpressing *FOXP1/HADC7*-. n = 3. Data shown are representative of 3 independent replications. **c** SA-β-gal staining at passage 5 of FOXP1/HDAC7 retroviral overexpressing hMSCs. Right panel, quantification of percentage of SA-β-gal-positive cells. Bar, 200 μm. **d** Immunofluorescence of LAP2 (red) and DAPI (blue) of passage 5 MSCs from mice (n = 3) as indicated. Right panel, quantification of LAP2-positive cells. Bar, 100 μm. **e** Immunofluorescence of γH2AX (Green) and DAPI (blue) for passage 5 MSCs from mice (n = 3) as indicated. Right panel, quantification of γH2AX-positive cells. Bar, 100 μm. *, *P* ≤ 0.05; **, *P* ≤ 0.01; ***, *P* ≤ 0.001; ns, non-significant

To address the mechanism of proliferative loss, senescence-associated β-galactosidase (SA-β-gal) staining was performed on MSCs at passage 5 (P5) of cultures. As shown in Fig. 5b, this hypothesis was validated, as premature senescence of MSCs from double KO mice was enhanced. This conclusion was corroborated by the observed increase in *p16* expression (Fig. 4b, e), by the relative decrease in the frequency of lamina-associated polypeptide 2 (LAP2)-positive cells (Fig. 4c, e), and by the increased numbers of γH2AX-positive cells—both canonical markers of DNA damage in senescent cells (Fig. 4d).

Taking with the impact of FOXP1/HDAC7 deficiency on MSC differentiation and self-renewal, we suggest that HDAC7 cooperatives with FOXP1 and facilitates FOXP1 transcription during bone mass maintenance, possibly through promotion of MSC self-renewal and prevention of premature senescence.

### Overexpression of FOXP1/HDAC7 in human MSCs augments their replicative capacity

Given the necessity of both FOXP1 and HDAC7 in MSC self-renewal, we tested whether overexpression of *FOXP1* and HDAC7 might attenuate senescence in human MSCs. Primary human MSCs (hereafter termed hMSC) [44] were infected with retroviral *pMSCV-FOXP1* and/or *pMSCV-HDAC7*. Overexpression efficiency was assessed by qPCR and western blot (Additional file 1: Fig. S4a, b). Of note, overexpression of HADC7 decreased the acetylation of FOXP1 as compared to controls (Additional file 1: Fig. S4c). As judged by the population doubling time of MSCs during in vitro passages, overexpression of *HDAC7* had little effect on MSC replicative capacity, whereas *FOXP1* overexpression clearly promoted MSC expansion (Fig. 5a). Of note, dual overexpression of *FOXP1* and *HDAC7* had an additive effect on MSC expansion capacity (Fig. 5a). For example, in passage 5 of MSC dual overexpressing cultures, significant downregulation of the *p16* and the *p21* cell cycle inhibitors were observed (Fig. 5b). Within the same passage, these MSCs also showed reduction in SA-β-gal staining (Fig. 5c), increased LAP2-positive cells and reduced γH2AX-positive DNA damage percentages (Fig. 5d and e). In addition, tri-lineage (osteogenic, chondrogenic and adipogenic) differentiation assays revealed that overexpression of *FOXP1* and *HDAC7* promoted MSC osteogenic capacity while suppressing adipogenic potency to a similar extent as that of single *FOXP1* overexpression (Additional file 1: Fig. S4d, e). Of note, overexpression of *HDAC7* and *FOXP1* had little impact on chondrogenic differentiation, as revealed by Alcian blue staining and qPCR analysis with chondrogenic markers (Additional file 1: Fig. S4f).

These observations indicate that overexpression of *FOXP1* and *HDAC7* additively augments MSC replicative capacity and attenuates their cellular senescence in vitro.

### Deacetylation of FOXP1^T172^ is crucial for self-renewal potency of hMSCs

Given that deacetylation of FOXP1 by HDAC7 is crucial for MSC self-renewal potency, we set out to determine the FOXP1 residues crucial for this process. As shown by our mass spectral (MS) analysis (Additional file 1: Fig. S1b), an array of amino acids composed of lysine and threonine was detected in acetylated clusters within the conserved leucine zipper (LZ), zinc finger (ZF) and forkhead (Fkh) domains. Recurrent variants within these domains are frequently observed in cohorts of neurodevelopmental disorders [45] as well as in cardiovascular, immunologic and several cases of malignancy [35, 46, 47], highlighting the susceptibility to mutation within these regions. Of note, there are two residues (T172 and T273) within the N-terminal QRF region, which lie distant, both linearly and three dimensionally [48], from the above “core conserved,” singly acetylated residues.

To address this issue, we constructed a FOXP1 cDNA expression vector carrying either T^172G^ or T^273G^ point mutation. The effect of these substitution mutations was then evaluated on FOXP1 transactivation ability by employing luciferase reporters *p16-Luc* and *RBPjκ-Luc* in C3H10T1/2 cells (Additional file 1: Fig. S5a, b). FOXP1^T172G^ catalyzed higher levels of *p16*-driven transcription repression than did the FOXP1^T273G^. Both WT and FOXP1^T172G^ mildly alleviated repression of *RBPjκ*-driven transactivation.

**Fig. 6:**
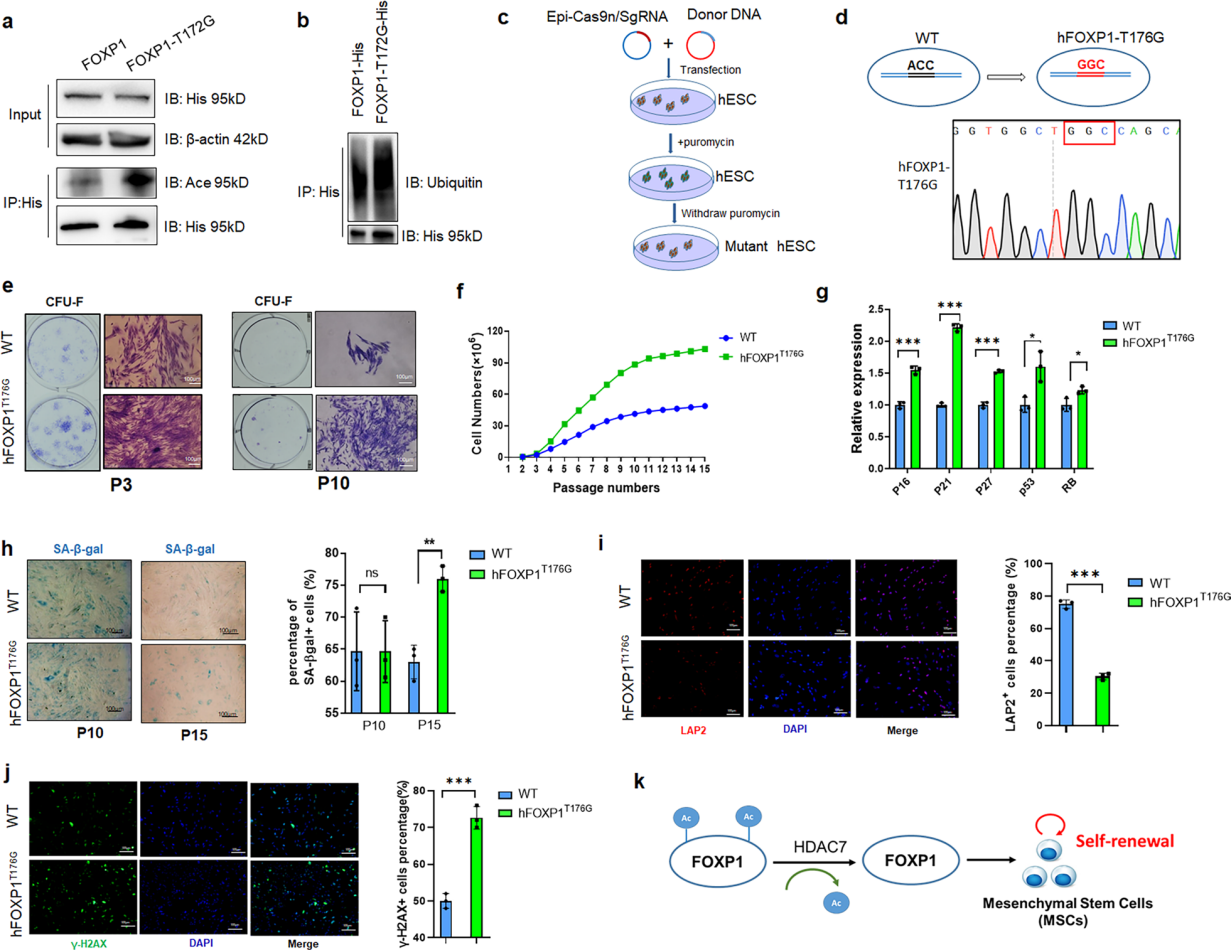
hFOXP1^T176G^ mutation potentiates the replicative capacity of hMSCs. **a** A murine FOXP1-T172G substitution mutant transfected into 293 T cells is enriched in global acetylation relative to FOXP1 wild type (WT) control. Proteins were pulled down via their His-tags by anti-His, fractionated on SDS-PAGE and then blotted with either pan anti-Acetyl-Lysine antibody (Ace) or for loading control antibodies specific for HA tag or for β-actin. **b** The ubiquitination levels of FOXP1-T172G were increased as compared to FOXP1 controls following transfection and fractionation in 293 T cells as described in panel A. **c** Schematic diagram depicting the strategy employed for hFOXP1^T176G^ engineering in human Embryonic Stem Cells (hESCs) via transfection with episomal Cas9n/sgRNA and donor vectors. **d** Validation of hFOXP1^T176G^-engineered hESCs by Sanger sequencing. The mutated leucine to glycine codon (GGC) is boxed in red (lower panel) relative to the wild type codon (ACC) shown in the left panel as black. **e** Crystal purple staining identifies CFU-F-positive colonies of hMSCs at passages P3 and P10. hMSCs shown were directly induced from hFOXP1^T176G^-engineered hESCs. Bar, 100 μm. **f** Population doubling curve of hFOXP1^T176G^-engineered hMSCs. Results shown are representative of 3 independent measurements. **g** Q-PCR analysis of cellular senescence as indicated by levels of cell cycle (*p16, p21, p27*) and tumor repressor (*p53, Rb*) transcript levels within hMSCs at P15. **h** SA-β-gal staining identifies cellular senescence of hFOXP1^T176G^-engineered hMSCs at P10 and P15. Data are representative of 3 independent measurements. Bar, 100 μm. **i** DNA damage as measured in hMSCs at P15 by immunofluorescence of Lamina-associated polypeptide 2 (LAP2) (left panel). Right panel, quantification of γH2AX-positive cells. n = 3. Bar, 100 μm. **j** Immunofluorescence of γH2AX in hMSCs at P15. Right panel is quantification of γH2AX-positive cells. Results shown are representative of 3 independent measurements. *, *P* ≤ 0.05; **, *P* ≤ 0.01; ***, *P* ≤ 0.001; ns, non-significant. Bar, 100 μm. **k** Schematic diagram showing that deacetylation of acetylation marks (blue dots) from FOXP1 by HDAC7 potentiates its effect on promoting self-renewal (indicated by the red arrow) of MSCs

To determine the consequences of FOXP1^T172G^ mutation on FOXP1 acetylation, we transfected pcDNA-FOXP1-T^172^-His or pcDNA-FOXP1-T^172G^-His into 293 T cells, harvested cell lysates via anti-His IP, and examined pan-acetylation by western blotting. As shown in Fig. 6a and b, FOXP1^T172G^ demonstrated significantly higher levels of both gross acetylation and ubiquitination than did FOXP1^T172^. These results, when combined with those of the MS and transcriptional analyses, confirmed that T172 was a critical site for acetylation potency.

To evaluate the impact of these point mutations on MSC self-renewal capacity, we generated an embryonic stem cell (ESC) line that expressed the *hFOXP1*^*T176G*^ point mutant through CRISPR/Cas9 engineering of a human ESC line (CCRM-hESC-22). Note that murine FOXP1^T172^ is equivalent to human FOXP1^T176^ due to modest isoform variation between the two species (Additional file 1: Fig. S5c, d). As illustrated in Fig. 6c, hESC cells were then transfected with episomal-Cas9n/donor DNA, screened with puromycin for one week, diluted as single cells/well, and selected with massive parallel PCR genotyping to obtain an ESC colony of the *hFOXP1*^*T176G*^ point mutant (Fig. 6c, d, Additional file 1: Fig. S5c, d). The *hFOXP1*^*T176G*^ ESC colony was further expanded and directly induced to transdifferentiate into the MSC lineage using a method previously described [25].

The self-renewal and replicative capacity of these transduced MSCs were then assessed by colony forming (CFU-F) and proliferation assays. As shown in Fig. 6e and f), *hFOXP1*^*T176G*^ MSCs displayed higher expansion capability in vitro as compared to control cells prior to passage 10 (P10). In addition, *hFOXP1*^*T176G*^ MSCs exhibited higher osteogenic and adipogenic potency at P10 (Additional file 1: Fig. S6). However, *hFOXP1*^*T176G*^ MSCs underwent premature cellular senescence at passage 15 (P15), as evidenced by their higher expression of cell cycle inhibitors (*p16/p21/p27*) and tumor suppressor (*p53/Rb)*transcripts (Fig. 6g), their elevated SA-β-gal staining (Fig. 6h) as well as their lower levels of LAP2-positivity and higher percentages of γH2AX positivity as compared to controls (Fig. 6i, j).

Collectively, these data demonstrate that a single point mutation at T176 within an acetylation site of human FOXP1 increased not only hMSC self-renewal potency but potentially led to precocious exhaustion of their replicative capacity.

## Discussion

In this study, we showed that the FOXP1 transcription factor bears multiple acetylation sites within several of its functional domains which are subject to deacetylation by histone deacetylase 7 (HDAC7). We employed an array of biochemical and genetic approaches that revealed that FOXP1 and HDAC7 cooperatively sustain bone marrow (BM) self-renewal of mesenchymal stem cells (MSCs) while diminishing their cellular senescence. For example, deacetylation of FOXP1 facilitated its stabilization, while potentiating MSC maintenance and protecting them from precocious senescence (Fig. 6k). Either ablation or dual-overexpression of FOXP1 and HDAC7 demonstrated their additive role in MSC replicative capability. Through genetic engineering, we created transgenic human embryonic stem cell-derived MSCs carrying a mutation within a conserved acetylation site (residue T172 in mice or T176 in humans) which dramatically augmented MSC expansion. These and additional findings revealed a mechanism by which deacetylation of FOXP1 controls MSC self-renewal and attenuates MSC cellular senescence.

While this posttranslational mechanism was unanticipated for FOXP1, a highly conserved paralog, FOXP3, also was reported to undergo HDAC7/9-mediated deacetylation by SIRT1 in regulatory T cells [49, 50]. However, the functional consequences of FOXP3 acetylation/deacetylation remain unclear.

The HDAC that regulates FOXP1 deacetylation may be context-dependent. We screened 18 HDACs comprising each of their homology classes to identify HDAC7 as the critical mediator for FOXP1 in MSCs. HDAC7 action was confirmed by co-IP both in vitro and in vivo. However, we cannot exclude the potential involvement of other HDACs in FOXP1 modification in other tissues. For instance, FOXO3 is deacetylated either by SIRT1 in T cells [11], but by several Class IIa HDACs in other cellular contexts [23].

During the course of the acetylation studies, we also observed that FOXP1 is ubiquitinated. We suggest that the acetylation of FOXP1 may prime its ubiquitination. MG132, a traditional ubiquitin-dependent degradation inhibitor, appeared to stabilize FOXP1 protein to an extent comparable to that of HDAC7. Importantly, deacetylation by HDAC7 was observed to particularly disrupt FOXP1 polyubiquitination at several sites conceivably to protect FOXP1 from proteasome-mediated degradation.

We observed that HDAC7 was most likely to interact with central and C-terminal domains of FOXP1. The central deacetylated residue for HDAC7 (T172 in mice and T176 in humans) resides within a relatively uncharacterized region termed the poly-glutamine- (or poly-Q)-rich domain. This region carries a run of 25–28 consecutive glutamines and is heavily glutamine-enriched for another ~ 100 residues (Additional file 1: Fig. S1a). Although structurally disordered [48], the Q-rich region is conserved in each of the 4 FOXP1 orthologs.

We also suspect that differential acetylation among the numerous sites in FOXP1 may cooperate with one other. For example, when FOXP1 T172 was mutated to G, neither total acetylation nor ubiquitination levels were decreased, but conversely, were increased (Fig. 6a, b). While we deem the potential interaction among acetylation and ubiquitination sites of FOXP1 an interesting topic, to further pursuit requires development of modification site-specific antibodies.

FOXP1 and HDAC7 cooperate in controlling MSC self-renewal, but have independent roles in regulating MSC differentiation potency, particularly in osteogenesis. As we previously reported [34], loss of FOXP1 in MSCs impairs their osteogenic potency at the expense of adipogenic differentiation. In contrast, HDAC7 deletion augments MSC-mediated osteoblast differentiation, possibly due to perturbation of its repressive interaction with RUNX2 [43]. Consequentially, the *FOXP1/HDAC7* double cKO, as compared to each single KO, displayed higher potency at an early development stage, but not at a more mature stage of osteogenic differentiation. MSCs with *FOXP1/HDAC7* double deficiency showed higher expression levels of early osteogenic markers (*Alp, Runx2, Osterix*), but not mature osteoblast markers (*DMP1* and *SOST*), as compared to MSCs with single *HDAC7* depletion (Fig. S3a, b). *HDAC7*^+/−^ KO MSCs also exhibited higher adipogenic potency, but the mutation had little effect on chondrogenesis. On the contrary, overexpression of HDAC7 and FOXP1 in hMSCs augmented their osteogenic capacity as compared to that with single FOXP1 overexpression (Additional file 1: Fig. S4d, e). Therefore, FOXP1 and HDAC7 exert differential effects on MSC self-renewal and differentiation, possibly through different pathways.

The development of site-specific gene editing based on CRISPR/Cas9 technology has led to an array of longevity genes engineered to extend lifespan, or to promote tissue regeneration. For instance, when two FOXO3 phosphorylation sites were replaced with alanine (S253A, S315A) via targeted gene editing, the nucleocytoplasmic shuttling of FOXO3 was stabilized within the nucleus where it promoted vascular protection and regeneration of MSCs [24, 25]. Here, we identified murine FOXP1 T172 as a candidate site for human gene engineering, as its mutation enhances MSC self-renewal. Curiously, a low frequency missense variation T176N in human FOXP1 (the equivalent position of murine FOXP1 T172) was detected in the Genome Aggregation Database (*gnomAD*) (Additional file 1: Fig. S7a). In addition, the T172 site is conserved in most mammalian genomes, other than zebrafish (Additional file 1: Fig. S7b). This suggests that variants within FOXP1 T172 are relatively safe and unsusceptible to developmental disorders.

Another reason we chose to focus our engineering on an acetylation site, such as T172, was that only a few mutations have been reported in the N-terminal, Q-rich region of FOXP1 in human diseases. The LZ and ZF domains of FOXP1 are crucial for dimerization, and the Fkh domain is responsible for DNA binding [51]. Mutations or variants within those conserved domains are susceptible to neurodevelopmental disorders, which are characterized by intellectual disability, autism, and language impairment [45]. For instance, a battery of missense mutations, including within the Fkh domain of human FOX1, has been detected in cohorts with intellectual syndrome [45, 52]. A P568S mutation near the C-terminus of FOXP1 is associated with congenital heart defects [53]. In contrast, a P215A substitution within the FOXP1 Q-rich region was deemed unlikely to underlie developmental verbal dyspraxia (DVD) [54].

Human MSCs of different origins have been widely employed to treat various diseases or promote tissue regeneration. At last citing, more than one thousand clinical trials with MSCs have been registered (https://www.clinicaltrials.gov/). The increased numbers of those clinical trials generate huge demands for MSCs of high replicative capacity and low risk of tumorigenesis. FOXP1 has been reported to be associated several human malignancies, including endometrial cancer, lung cancer, head and neck cancer, prostate cancer, renal cell carcinoma, ovarian carcinoma, osteosarcoma, hepatocellular carcinoma and B cell lymphoma [33, 47, 55–60].

In this regard, we demonstrated that FOXP1 is essential for tumorigenesis of the more aggressive form of Diffuse Large B-Cell Lymphoma, termed Activated B Cell (ABC-DLBCL)[60]. We find it interestingly in the present context that ABC-DLBCL tumors display high enrichment of acetylated lysine relative to the quite modest levels observed in the more indolent Germinal Center (GC)-DLBCL (Data not shown).

FOXP1^T176G^-engineered hMSCs retain high expansive capability during early passages, and precocious cellular senescence at later stages. After a vigorous phase of replication prior to passage P10, FOXP1-engineered hMSCs reached stationary phase at ~ P15. At this stage, higher expression of cell cycle inhibitor transcripts (*p16, p21, p27*) and tumor suppressor transcripts (*p53, Rb*) were observed. Thus, the likelihood for tumorigenesis induction within FOXP1^T176G^-engineered hMSCs is quite low.

## Conclusion

In summary, we have unraveled the deacetylation mechanism mediated on FOXP1 by HDAC7 which is critical to the maintenance of MSC self-renewal potency and safeguard them from senescence. The studies summarized above allow us to predict that FOXP1-T172 will provide a safe and promising approach for bioengineering highly replicative MHCs. Our study sheds new light onto the strategy utilizing FOXP1-engineered stem cells for large-scale MSC in vitro expansion in the clinical setting.

## Supplementary Information

**Additional file 1:** Supplemental figures and figure legends. Supplemental Materials and Methods.

## Data Availability

All original data were available upon request to correspondent author. The raw MS data of FOXP1 protein modifications were available from PeptideAtlas (dataset Identifier: PASS03792).

## References

[1] Chan CK, Seo EY, Chen JY (2015). Identification and specification of the mouse skeletal stem cell. Cell.

[2] Chan CKF, Gulati GS, Sinha R (2018). Identification of the human skeletal stem cell. Cell.

[3] Debnath S, Yallowitz AR, McCormick J (2018). Discovery of a periosteal stem cell mediating intramembranous bone formation. Nature.

[4] Duchamp de Lageneste O, Julien A, Abou-Khalil R (2018). Periosteum contains skeletal stem cells with high bone regenerative potential controlled by Periostin. Nat Commun..

[5] Ambrosi TH, Longaker MT, Chan CKF (2019). A revised perspective of skeletal stem cell biology. Front Cell Dev Biol.

[6] Ding L, Morrison SJ (2013). Haematopoietic stem cells and early lymphoid progenitors occupy distinct bone marrow niches. Nature.

[7] Ren G, Zhang L, Zhao X (2008). Mesenchymal stem cell-mediated immunosuppression occurs via concerted action of chemokines and nitric oxide. Cell Stem Cell.

[8] Le Blanc K, Rasmusson I, Sundberg B (2004). Treatment of severe acute graft-versus-host disease with third party haploidentical mesenchymal stem cells. Lancet.

[9] Liu Y, Wang L, Kikuiri T (2011). Mesenchymal stem cell-based tissue regeneration is governed by recipient T lymphocytes via IFN-gamma and TNF-alpha. Nat Med.

[10] Collado M, Blasco MA, Serrano M (2007). Cellular senescence in cancer and aging. Cell.

[11] Brunet A, Sweeney LB, Sturgill JF (2004). Stress-dependent regulation of FOXO transcription factors by the SIRT1 deacetylase. Science.

[12] Mouchiroud L, Houtkooper RH, Moullan N (2013). The NAD(+)/sirtuin pathway modulates longevity through activation of mitochondrial UPR and FOXO signaling. Cell.

[13] Yoon DS, Choi Y, Jang Y (2014). SIRT1 directly regulates SOX2 to maintain self-renewal and multipotency in bone marrow-derived mesenchymal stem cells. Stem Cells.

[14] Zainabadi K (2018). The variable role of SIRT1 in the maintenance and differentiation of mesenchymal stem cells. Regen Med.

[15] Yuan HF, Zhai C, Yan XL (2012). SIRT1 is required for long-term growth of human mesenchymal stem cells. J Mol Med (Berl).

[16] Pan H, Guan D, Liu X (2016). SIRT6 safeguards human mesenchymal stem cells from oxidative stress by coactivating NRF2. Cell Res.

[17] Denu RA (2017). SIRT3 enhances mesenchymal stem cell longevity and differentiation. Oxid Med Cell Longev.

[18] Zhang DY, Zhang CF, Fu BC (2018). Sirtuin3 protects aged human mesenchymal stem cells against oxidative stress and enhances efficacy of cell therapy for ischaemic heart diseases. J Cell Mol Med.

[19] Diao Z, Ji Q, Wu Z (2021). SIRT3 consolidates heterochromatin and counteracts senescence. Nucleic Acids Res.

[20] Bi S, Liu Z, Wu Z (2020). SIRT7 antagonizes human stem cell aging as a heterochromatin stabilizer. Protein Cell.

[21] Morris BJ, Willcox DC, Donlon TA (2015). FOXO3: a major gene for human longevity–a mini-review. Gerontology.

[22] van Heemst D (2010). Insulin, IGF-1 and longevity. Aging Dis.

[23] Mihaylova MM, Vasquez DS, Ravnskjaer K (2011). Class IIa histone deacetylases are hormone-activated regulators of FOXO and mammalian glucose homeostasis. Cell.

[24] Lei J, Wang S, Kang W (2021). FOXO3-engineered human mesenchymal progenitor cells efficiently promote cardiac repair after myocardial infarction. Protein Cell.

[25] Yan P, Li Q, Wang L (2019). FOXO3-engineered human ESC-derived vascular cells promote vascular protection and regeneration. Cell Stem Cell.

[26] Azagra A, Roman-Gonzalez L, Collazo O (2016). In vivo conditional deletion of HDAC7 reveals its requirement to establish proper B lymphocyte identity and development. J Exp Med.

[27] Gabut M, Samavarchi-Tehrani P, Wang X (2011). An alternative splicing switch regulates embryonic stem cell pluripotency and reprogramming. Cell.

[28] Naudin C, Hattabi A, Michelet F (2017). PUMILIO/FOXP1 signaling drives expansion of hematopoietic stem/progenitor and leukemia cells. Blood.

[29] Feng X, Wang H, Takata H (2011). Transcription factor Foxp1 exerts essential cell-intrinsic regulation of the quiescence of naive T cells. Nat Immunol.

[30] Fu NY, Pal B, Chen Y (2018). Foxp1 is indispensable for ductal morphogenesis and controls the exit of mammary stem cells from quiescence. Dev Cell.

[31] Leishman E, Howard JM, Garcia GE (2013). Foxp1 maintains hair follicle stem cell quiescence through regulation of Fgf18. Development.

[32] Pearson CA, Moore DM, Tucker HO (2020). Foxp1 regulates neural stem cell self-renewal and bias toward deep layer cortical fates. Cell Rep.

[33] Zhang Y, Zhang S, Wang X (2012). Prognostic significance of FOXP1 as an oncogene in hepatocellular carcinoma. J Clin Pathol.

[34] Li H, Liu P, Xu S (2017). FOXP1 controls mesenchymal stem cell commitment and senescence during skeletal aging. J Clin Invest.

[35] Kim JH, Hwang J, Jung JH (2019). Molecular networks of FOXP family: dual biologic functions, interplay with other molecules and clinical implications in cancer progression. Mol Cancer.

[36] Hu H, Wang B, Borde M (2006). Foxp1 is an essential transcriptional regulator of B cell development. Nat Immunol.

[37] Greenbaum A, Hsu YM, Day RB (2013). CXCL12 in early mesenchymal progenitors is required for haematopoietic stem-cell maintenance. Nature.

[38] Fernandez A, Morin M, Munoz-Santos D (2020). Simple protocol for generating and genotyping genome-edited mice With CRISPR-Cas9 reagents. Curr Protoc Mouse Biol.

[39] Jiang C, Cai L, Huang B (2013). Normal human embryonic stem cell lines were derived from microsurgical enucleated tripronuclear zygotes. J Cell Biochem.

[40] LI Zhenzhen QL, NING Song, CUI uugui, MA Xiang, LIU Jiayin. Effects of hyperandrogen on human embryonic stem cells induced to differentiate into hypothalamic neural progenitors. J Nanjing Med Univ*.* 2020:11.

[41] Park SY, Kim JS (2020). A short guide to histone deacetylases including recent progress on class II enzymes. Exp Mol Med.

[42] Chang S, Young BD, Li S (2006). Histone deacetylase 7 maintains vascular integrity by repressing matrix metalloproteinase 10. Cell.

[43] Jensen ED, Schroeder TM, Bailey J (2008). Histone deacetylase 7 associates with Runx2 and represses its activity during osteoblast maturation in a deacetylation-independent manner. J Bone Miner Res.

[44] Liu H, Jiang C, La B (2021). Human amnion-derived mesenchymal stem cells improved the reproductive function of age-related diminished ovarian reserve in mice through Ampk/FoxO3a signaling pathway. Stem Cell Res Ther.

[45] Siper PM, De Rubeis S, Trelles MDP (2017). Prospective investigation of FOXP1 syndrome. Mol Autism.

[46] Bray F, Ferlay J, Soerjomataram I (2018). Global cancer statistics 2018: GLOBOCAN estimates of incidence and mortality worldwide for 36 cancers in 185 countries. CA Cancer J Clin.

[47] Koon HB, Ippolito GC, Banham AH (2007). FOXP1: a potential therapeutic target in cancer. Expert Opin Ther Targets.

[48] Chu YP, Chang CH, Shiu JH (2011). Solution structure and backbone dynamics of the DNA-binding domain of FOXP1: insight into its domain swapping and DNA binding. Protein Sci.

[49] Kwon HS, Lim HW, Wu J (2012). Three novel acetylation sites in the Foxp3 transcription factor regulate the suppressive activity of regulatory T cells. J Immunol.

[50] Li B, Samanta A, Song X (2007). FOXP3 interactions with histone acetyltransferase and class II histone deacetylases are required for repression. Proc Natl Acad Sci U S A.

[51] Li S, Weidenfeld J, Morrisey EE (2004). Transcriptional and DNA binding activity of the Foxp1/2/4 family is modulated by heterotypic and homotypic protein interactions. Mol Cell Biol.

[52] Hamdan FF, Daoud H, Rochefort D (2010). De novo mutations in FOXP1 in cases with intellectual disability, autism, and language impairment. Am J Hum Genet.

[53] Chang SW, Mislankar M, Misra C (2013). Genetic abnormalities in FOXP1 are associated with congenital heart defects. Hum Mutat.

[54] Vernes SC, MacDermot KD, Monaco AP (2009). Assessing the impact of FOXP1 mutations on developmental verbal dyspraxia. Eur J Hum Genet.

[55] Choi EJ, Seo EJ, Kim DK (2016). FOXP1 functions as an oncogene in promoting cancer stem cell-like characteristics in ovarian cancer cells. Oncotarget.

[56] Bates GJ, Fox SB, Han C (2008). Expression of the forkhead transcription factor FOXP1 is associated with that of estrogen receptor-beta in primary invasive breast carcinomas. Breast Cancer Res Treat.

[57] Li H, Han X, Yang S (2021). FOXP1 drives osteosarcoma development by repressing P21 and RB transcription downstream of P53. Oncogene.

[58] Ackermann S, Kocak H, Hero B (2014). FOXP1 inhibits cell growth and attenuates tumorigenicity of neuroblastoma. BMC Cancer.

[59] Walker MP, Stopford CM, Cederlund M (2015). FOXP1 potentiates Wnt/beta-catenin signaling in diffuse large B cell lymphoma. Sci Signal..

[60] Dekker JD, Park D, Shaffer AL (2016). Subtype-specific addiction of the activated B-cell subset of diffuse large B-cell lymphoma to FOXP1. Proc Natl Acad Sci U S A.

