# Supplementary material for: Deacetylation of FOXP1 by HDAC7 potentiates self-renewal of mesenchymal stem cells
Source: Stem Cell Res Ther. 2023 Jul 28;14:188. doi: 10.1186/s13287-023-03376-7 (PMC10385979; doi:10.1186/s13287-023-03376-7)
Supplement: Supplementary file 1 — Additional file 1: Supplemental figures and figure legends. Supplemental Materials and Methods. [file 13287_2023_3376_MOESM1_ESM.docx]

Supplemental figures and figure legends

**
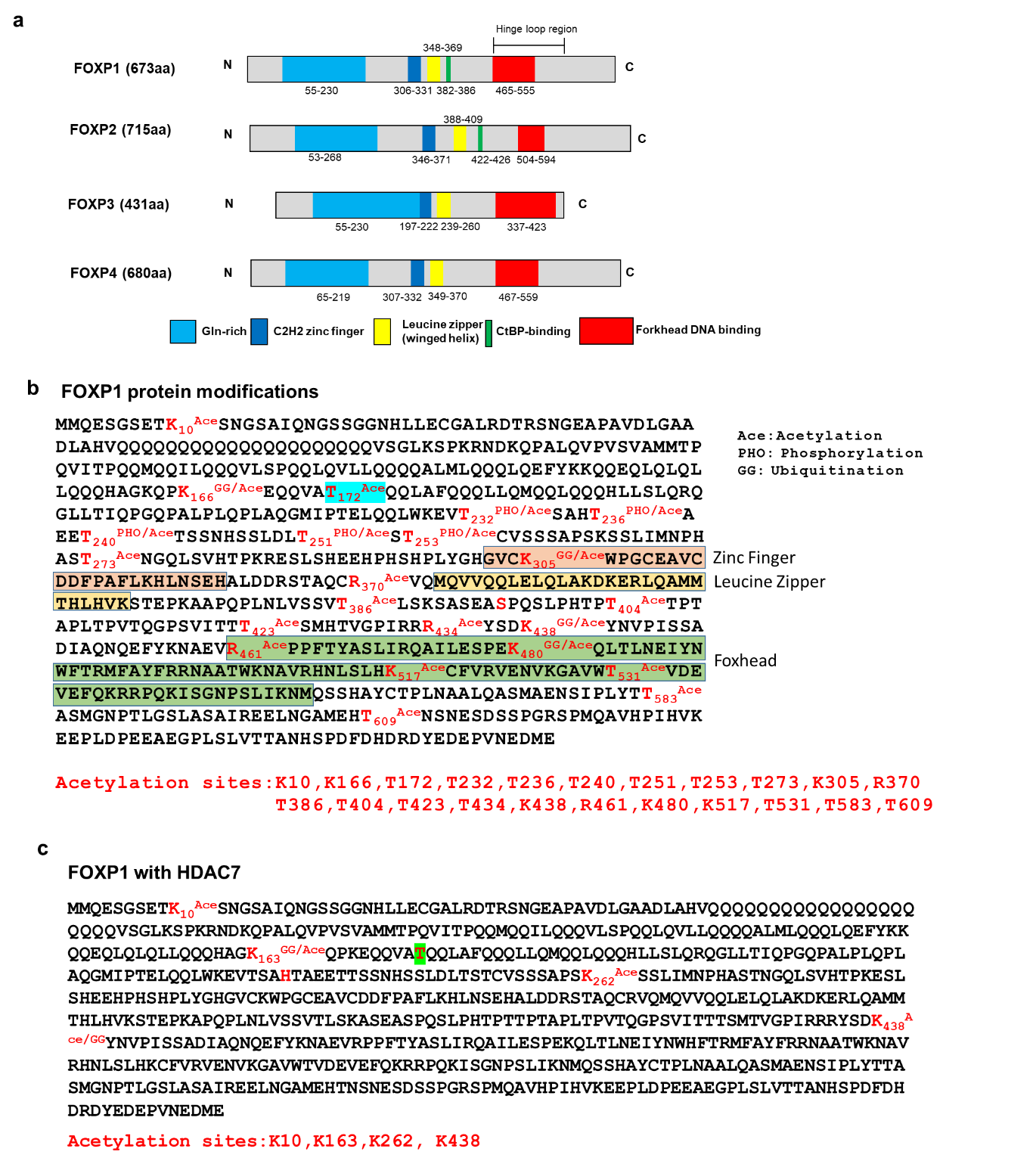
**

**Fig. S1** Acetylation sites within murine FOXP1.

1. Diagram depicting the structure of FOXP family proteins of FOXP1-4. Five highly conserved domains are detected in all the four proteins as indicated with different colored boxes: Gln-rich domain (light blue); C2H2 zinc finger domain (deep blue); Leucine zipper domain (yellow); CtBP binding domain (green); Foxhead DNA binding domain (red).
2. Mass spectrum identification of the potential acetylation sites in murine FOXP1 following its enrichment by anti-His tag-mediated pull-down from 293T cells transfected with pcDNA-FOXP1-His. Acetylation-only (Ace) amino acids included K10, T172, T273, R370, T386, T404, T423, R434, R461, K517 and T531. Several sites including K166, T232, T236, T240, T250, T253, K305, K438 and K480 were detected with dual phosphorylation/acetylation (PHO/Ace) or ubiquitination/acetylation (GG/Ace) modifications. FOXP1 domain residues are colored as Zinc Finger (tan), Leucine Zipper (yellow) and Foxhead (green).
3. Mass spectrum identification of acetylation sites in murine FOXP1 upon the presence of HDAC7. FOXP1 protein was enriched from 293T cells transfected with pcDNA-FOXP1-His and pcDNA-HDAC7-Flag. Acetylation was detected at amino acids K10, K163, K262, and K438. This indicated a profoundly reduction of acetylation level within FOXP1 upon HDAC7 action.


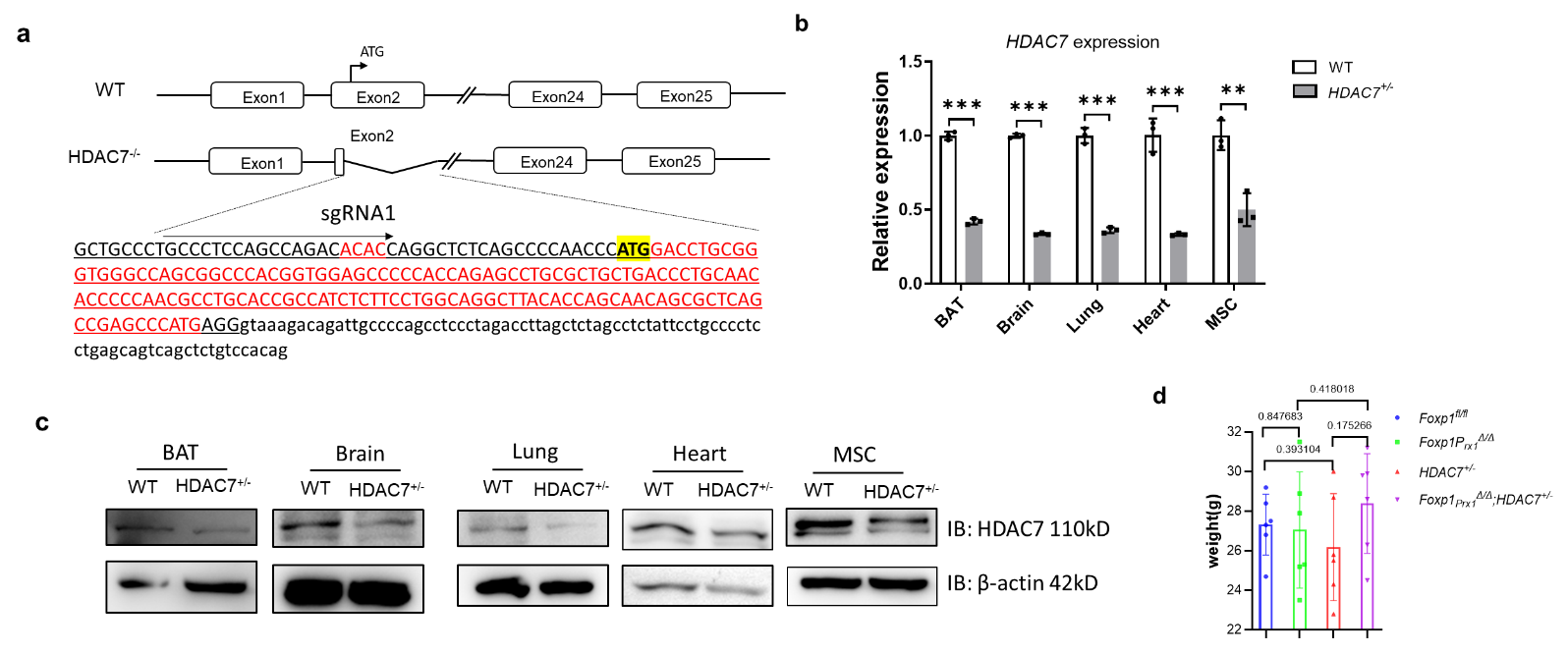


**Fig. S2** Generation and characterization of *HDAC7^+/-^* mice

1. Schematic diagram depicting the gene structure of the *HDAC7* null allele that was generated by CRISPR/Cas9-mediated gene editing. A 142 bp-fragment (red) spanning the ATG initiation codon in exon 2 was deleted. The sgRNA1 location is indicated with an arrow.
2. Assessment of *HDAC7* transcript expression by qPCR in brown adipose tissues (BAT), brain, lung, heart and MSCs derived from the bone marrow (BM). *, *P*≤0.05; **, *P*≤0.01; ***, *P*≤0.001; ns, nonsignificant.
3. Assessment of HDAC7 expression by western blot in the indicated tissues.

**d** Statistics of body weight of 3-month-old male mice (n=3) with genotypes: *Foxp1^fl/fl^*, *Foxp1_Prx1_^∆/∆^*, *HDAC7^+/-^*, and *Foxp1_Prx1_^∆/∆^;HDAC7^+/-^*.


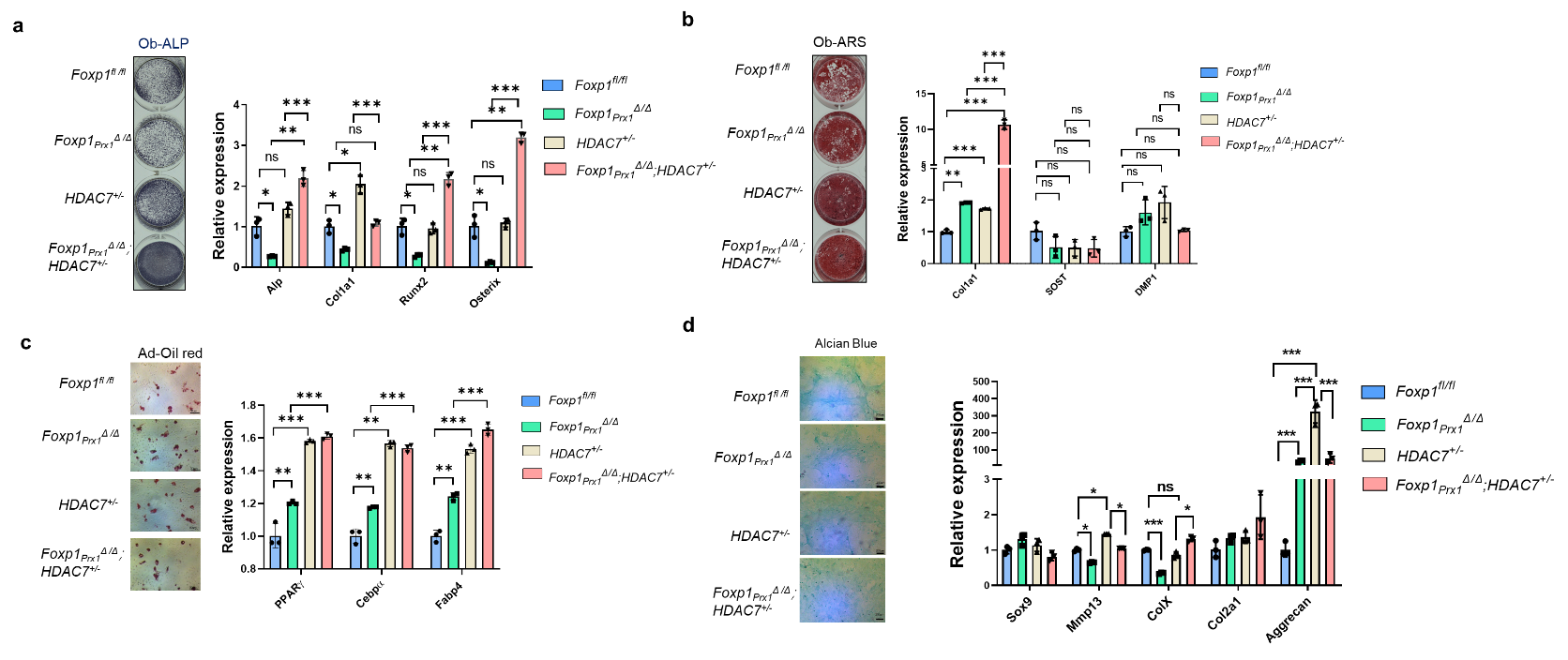


**Fig. S3** Tri-lineage differentiation of MSCs from *Foxp1/HDAC7* knockout mice.

**a, b** ALP/ARS staining (left panel) and qPCR analysis (right panel) for osteogenic differentiation of MSCs from BM of 3-month-old mice (n=3) with genotypes: *Foxp1^fl/fl^*, *Foxp1_Prx1_^∆/∆^*, *HDAC7^+/-^*, and *Foxp1_Prx1_^∆/∆^;HDAC7^+/-^*.

**c** Oil red O staining and qPCR analysis of adipogenic differentiation of MSCs from BM of 3-month-old mice (n=3) of the indicated genotypes. Bar, 50μm.

**d** Alcian blue staining and qPCR analysis to assess osteogenic differentiation of MSCs from BM of 3-month-old mice (n=3). Bar, 200μm.

*, *P*≤0.05; **, *P*≤0.01; ***, *P*≤0.001; ns, non significant.


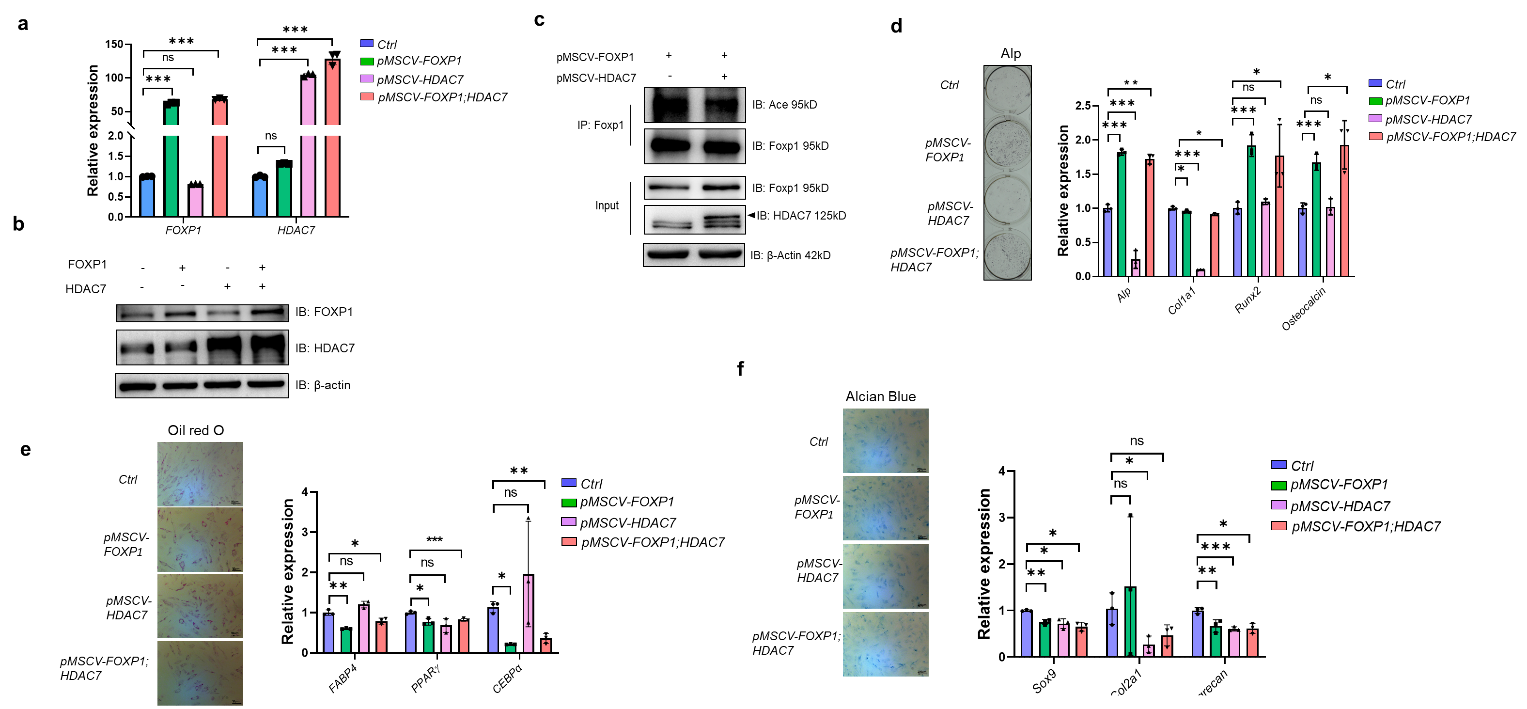


**Fig. S4** Tri-lineage differentiation capacity of hMSCs retrovirally overexpressing FOXP1 and HDAC7

**a, b** qPCR (a) and western blot (b) analyses for FOXP1 and HDAC7 expression in hMSCs transfected with retroviral *pMSCV-FOXP1* and/or *pMSCV-HDAC7;* hMSCs transfected with *pMSCV* empty vector were used as controls. Data are representative of 3 independent experiments.

**c** Evaluation of acetylation level of FOXP1 with western blot in immunoprecipitates from hMSCs lysates with FOXP1 and/or HDAC7 overexpression.

**d** ALP staining (left panel) and qPCR analysis (right panel) of osteogenic differentiation of hMSCs from (a). Bar, 50μm.

**e** Oil red O staining (left panel) and qPCR analysis (right panel) for adipogenic differentiation of hMSCs from (a). Bar, 200μm.

**f** Alcian blue staining (left panel) and qPCR analysis (right panel) for chondrogenic differentiation of hMSCs from (a).

*, *P*≤0.05; **, *P*≤0.01; ***, *P*≤0.001; ns, not significant.


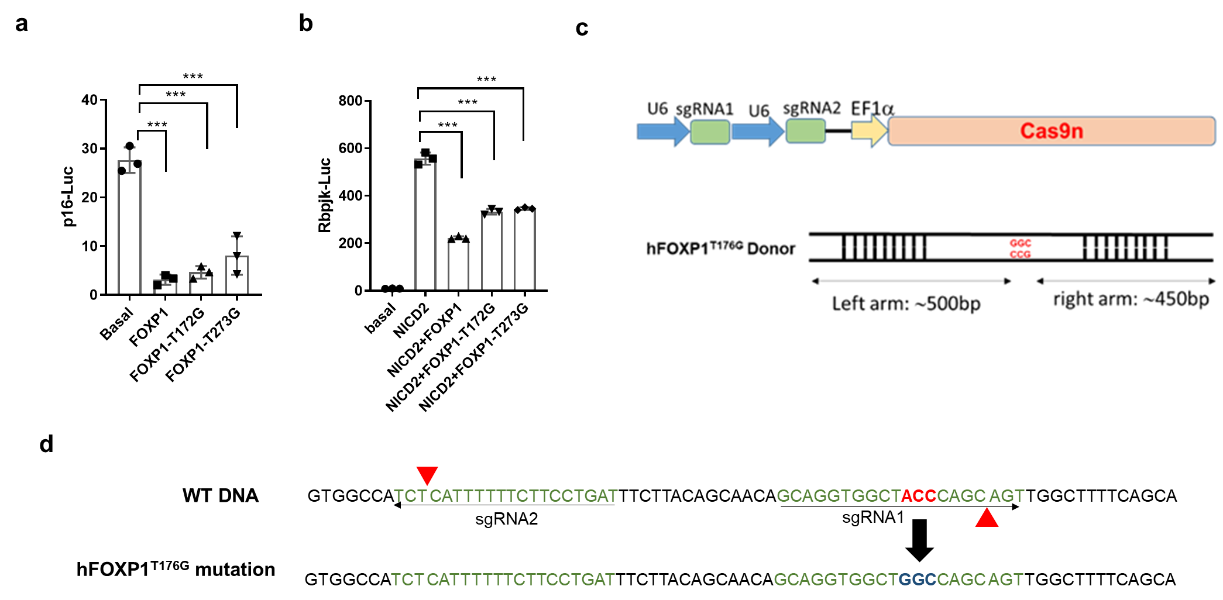


**Fig. S5** CRISPR/Cas9n constructs for generation of hFOXP1^T176G^ ESCs

**a, b** Assessment of the transactivation ability of FOXP1-T172G or FOXP1-273G mutant proteins by luciferase reporter assays. 293T cells were transfected with *p16*-Luciferase (*p16*-Luc) (a) or *Rbpjκ*-Luc vector (b) either with or without co-transfection with FOXP1 mutant plasmids as indicated. *, *P*≤0.05; **, *P*≤0.01; ***, *P*≤0.001; ns, nonsignificant.

**c** Diagrams depicting the gene structures of episomal Cas9n/sgRNA (upper panel) and hFOXP1^T176G^ donor DNA (lower panel). Two opposite sgRNAs1/2 (denoted in blue) were driven by the U6 promoter, and the Cas9n fragment (colored tan) were driven by the EF1α promoter.

**d** Genomic locations and sequences of sgRNA1/2 (green), the hFOXP1^T176G^ mutation site (ACC/GGC), and presumptive nick site (red arrow heads) by Cas9n.


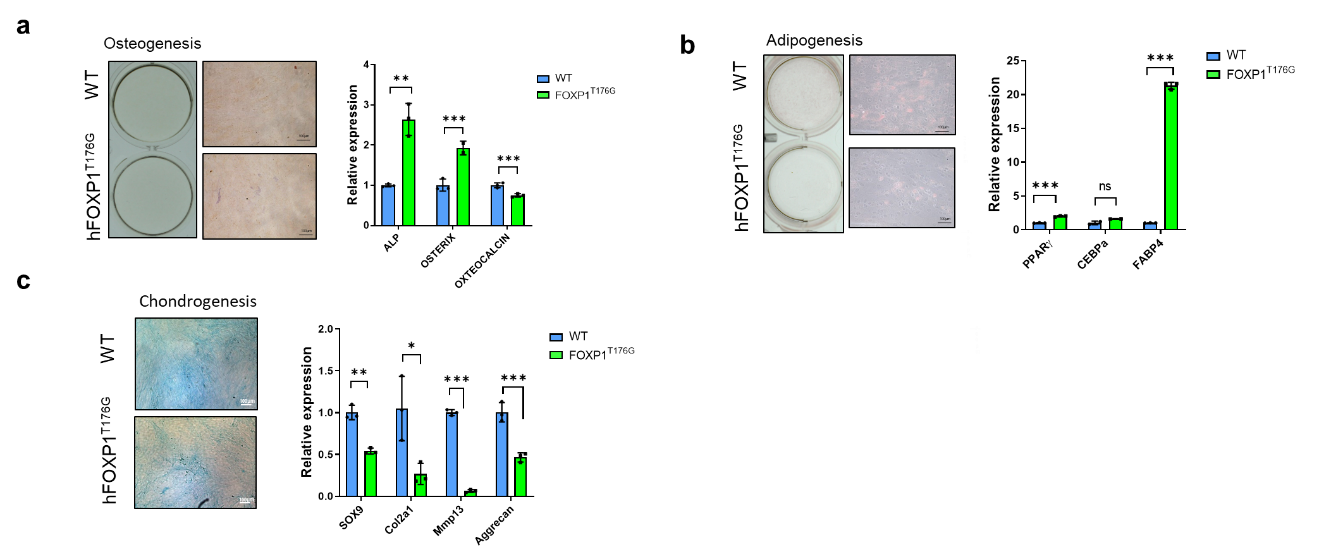


**Fig. S6** FOXP1^T176G^ hMSC tri-lineage differentiation capacity at P10

**a** ALP staining (left panel) and qPCR analysis (right panel) for osteogenic differentiation of hFOXP1^T176G^ hMSCs at P10.

**b** Oil red O staining (left panel) and qPCR analysis (right panel) for adipogenic differentiation of hFOXP1^T176G^ hMSCs at P10.

**c** Alcian blue staining (left panel) and qPCR analysis (right panel) for chondrogenic differentiation of hFOXP1^T176G^ hMSCs at P10.

*, *P*≤0.05; **, *P*≤0.01; ***, *P*≤0.001; ns, non significant.


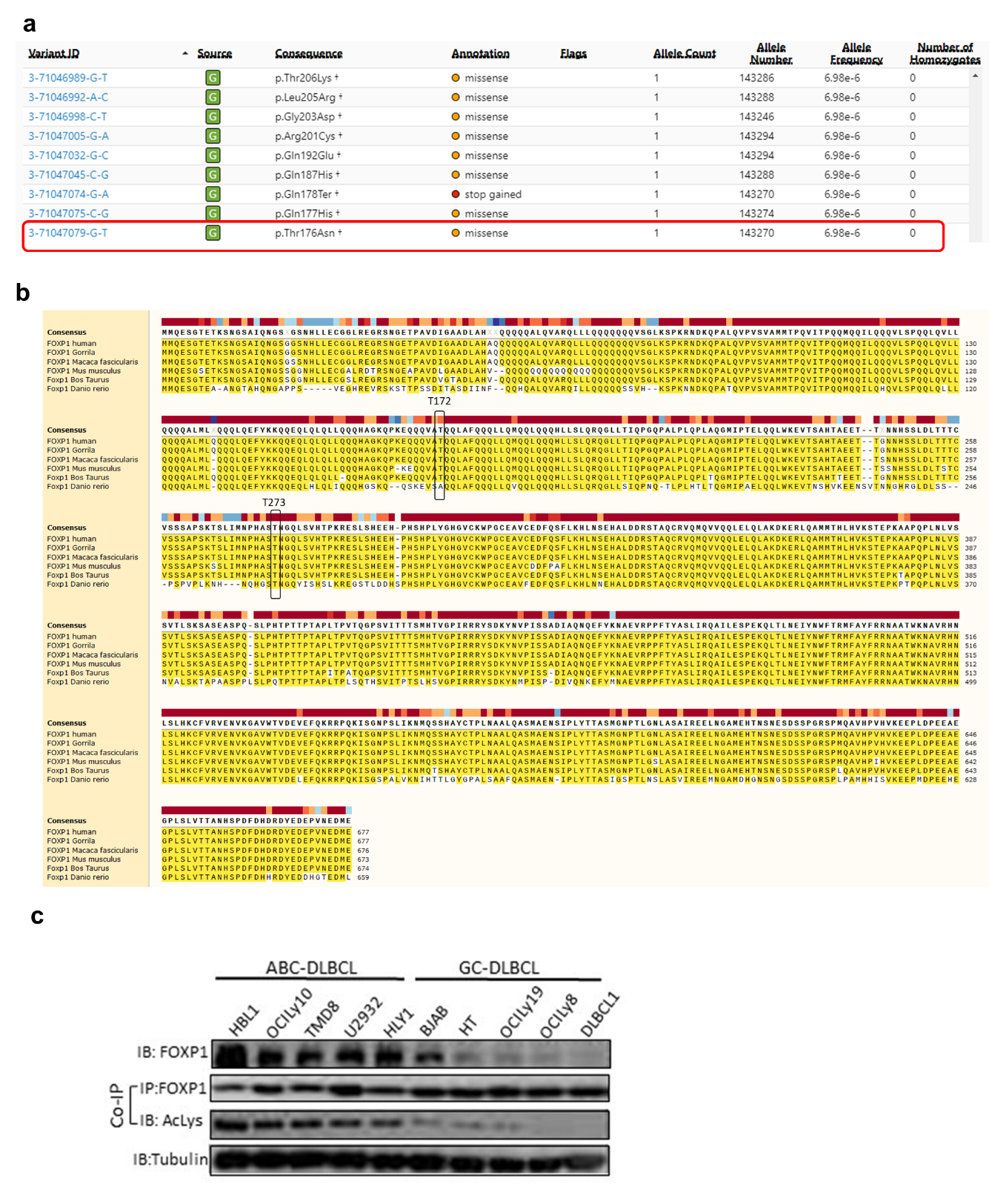


**Fig. S7** Amino acid variants of FOXP1 previously published and downloaded from the *gnomADv3* database

1. One rare missense variant of FOXP1 T176N was derived from ~1000 human sequences determined by genomic sequencing projects.
2. Murine FOXP1 T172 was conserved in mammalian genomes, but not in zebrafish. FOXP1 T273 was conserved in all genomes.

**Supplemental Materials and Methods**

**Evaluation of tri-lineage differentiation of MSCs.**

MSCs were cultured in either osteogenic medium (α-MEM with 10% FBS, 50μg/mL VC, 10mM β- glycerophosphoric acid, and 0.1μM dexamethasone) for 14-21 days, or in adipogenic medium (DMEM with 10% FBS, 10 μg/mL insulin, 1 μM dexamethasone, 0.5 mM IBMX, and 200 μM indometacin) for 21 days, or in chondrogenic medium (DMEM with 2% FBS, 0.1μM dexamethasone, 6.25μg/mL insulin, 6.25μg/mL transferrin, 10ng/mL TGF-β, 50μg/mL VC, and 1mM sodium pyruvate) for 21days. The resulting bone, adipocytes, and cartilage were assessed by staining with alkaline phosphate (ALP), ARS oil red O or Alcian blue, respectively.

**Generation of *FOXP1^T176G^* hESCs and Directed Differentiation into hMSCs.**

The epiCRISPRn system has been described preciously (Xie et al., 2017). In brief, an epiCRISPRn plasmid that contains two opposing sgRNA and Cas9n fragments and linearized donor DNA were transfected into the hESCs with Lipo8000 (Beyotime). 48 hours later, cells were selected by puromycin (0.2-1μg/mL) for 5~7 days. The GFP positive clones were selected microscopically and then passaged twice using medium supplemented with puromycin (Sigma, 0.2-1μg/mL). Genomic DNA was extracted from cells, converted to cDNA and the mutation was validated by Sanger sequencing.

Differentiation of hESCs into hMSCs was performed as previously described (Yan *et al.*, 2019). In brief, hESCs were cultured in hMSC differentiation medium (α-MEM supplemented with 10% FBS plus 10 ng/ml bFGF, 5 ng/ml TGFβ and 1% penicillin/streptomycin) for 10 days until fibroblast-like cells appeared. Then cells were passaged twice until all hESCs were absent.

**Mass Spectra Analysis**

The peptides then were analyzed with an EASY-nL 1200 system coupled with a Q Exactive plus mass spectrometer (Thermo Scientific, Bremen, Germany). Briefly, aliquots containing peptides were injected and separated over an analytical column (C18, 20cm×75μm, 3μm) using a 2 hr acetonitrile gradient in 0.1% formic acid at a flow rate of 300 nL/min. The mobile phase solvent (A) contained 0.1% formic acid and solvent B contained 80% acetonitrile plus 0.1% formic acid. Fractionation was achieved by a linear gradient of 2% to 6% solvent B over 0 to 2 min, 6% to 20% over 95 min, 20% to 32% over 95 to 107 min, 32% to 100% over 107 to 108 min and then held for 12 min. The mass spectrometer was set as one full MS scan followed by 20 MS/MS scans on the 20 most intense ions from the MS spectrum. In MS1, the precursor scan was acquired at 70,000 resolution in the orbitrap, with the AGC set to 3e6 and 50 ms maximum injection time. The HCD-MS2 spectra were acquired at 17,500 resolution in the orbitrap, with a collision energy of 28%, with the AGC set at 1e5 with an isolation width of 1.6 m/z, using a maximum injection time of 45ms. The peptide sequences were determined by searching MS/MS spectra against the Protein database using the Protein Discoverer (version 2.5, Thermo Scientific) software suite with a precursor ion mass tolerance of 10 ppm and fragment ion mass tolerance of 0.02 Da. Carbamidomethyl (C) was set as the fixed modification, oxidation (M), delaminated (NQ), Acetyl (N-terminus), Met-loss (M) and Met-loss+Acetyl (M) were set as the variable modifications. For modification of ubiquitination (K), +114.0429 Da was set as the variable modification for lysine, and a maximum of two missed cleavages of trypsin was chosen.

Supplemental Table 1. Oligos for genotyping (GT), qPCR

| primers | sequences |
| --- | --- |
| Mus-Cre-F-GT: | TTTCCCGCAGAACCTGAAGA |
| Mus-Cre-R-GT: | GGTGCTAACCAGCGTTTTCGT |
| Mus-Foxp1-F-GT: | CTCCTAGTCACCTTCCCCAGTGC |
| Mus-Foxp1-R-GT: | GAACACTGTCGAATGACCCTGC |
| Mus-HDAC7-F-GT: | CAGAGGATTCAGTAGTCTCTG |
| Mus-HDAC7-R-GT: | CCTTACTTCGCTTGCTCTTG |
| Mus-ALP-qPCR-F: | GCCTGGATCTCATCAGTATTTGG |
| Mus-ALP-qPCR-R: | GTTCAGTGCGGTTCCAGACAT |
| Mus-Col1a1-qPCR-F: | CCGGAAGAATACGTATCACC |
| Mus-Col1a1-qPCR-R: | ACCAGGAGGACCAGGAAGTC |
| Mus-Osx-qPCR-F: | CTCTCTGCTTGAGGAAGAAG |
| Mus-Osx-qPCR-R: | GTCCATTGGTGCTTGAGAAG |
| Mus-Runx2-qPCR-F: | CCGGGAATGATGAGAACTA |
| Mus-Runx2-qPCR-R: | ACCGTCCACTGTCACTTT |
| Mus-Osteocalcin-qPCR-F: | GTTCAGGGTGTGT-CGTCGAAC |
| Mus-Osteocalcin-qPCR-R: | TTTCGGCTCGACGGTCTCAAA |
| Mus-Sox9-qPCR-F: | AGGAAGCTGGCAGACCAGTA |
| Mus-Sox9-qPCR-R: | TCCACGAAGGGTCTCTTCTC |
| Mus-ColX-qPCR-F: | CCCCAAGACACAATACTTCATCC |
| Mus-ColX-qPCR-R: | ATGCCTTGTTCTCCTCTTACTGG |
| Mus-Aggrecan-qPCR-F: | CTGAGGAACAGGAGTTCGTCAA |
| Mus-Aggrecan-qPCR-R: | CGGAAGTCCCCTTCGATAGTC |
| Mus-Col2a1-qPCR-F: | GAGCAGCAAGAGCAAGGAAAA |
| Mus-Col2a1-qPCR-R: | TCGCCATAGCTGAAGTGGAA |
| Mus-p21-qPCR-F: | GAACATCTCAGGGCCGAAAAC |
| Mus-p21-qPCR-R: | CTGCGCTTGGAGTGATAGAA |
| Mus-p27-qPCR-F: | ACTAACCCGGGACTTGGAGA |
| Mus-p27-qPCR-R: | GAAATTCCACTTGCGCTGAC |
| Mus-p16-qPCR-F: | CTAGAGAGGATCTTGAGAAGAGGGC |
| Mus-p16-qPCR-R: | TAGTTGAGCAGAAGAGCTGCTACGT |
| Mus-β-actin-qPCR-F: | AACAGTCCGCCTAGAAGCAC |
| Mus-β-actin-qPCR-R: | CGTTGACATCCGTAAAGACC |
| Mus-HDAC7-qPCR-F: | CTTCCTGGCAGGCTTACACC |
| Mus-HDAC7-qPCR-R: | GCTCAAGAGTTCTGTAGGGAATAC |
| Mus-HDAC7-mus-sgRNA1 | TAATACGACTCACTATAGGGCCCTCCAGCCAGACACACCGTTTTAGAGCTAGAAATAGCAA |
| Mus-HDAC7-mus-sgRNA2 | TAATACGACTCACTATAGGAGCCCCAACCCATGGACCTG GTTTTAGAGCTAGAAATAGCAA |
| FOXP1^T176G^-GT-F | CAGTGACCATTCTCAGCATT |
| FOXP1^T176G^-GT-R | ATAGCACATGTTACATGGTATAAAA |
| FOXP1^T176G^-sgRNA1 | CGCTCTTCGCCGGCAGGTGGCTACCCAGCAGTGTTTTAGAGCTAGAAATAGCAA |
| FOXP1^T176G^-sgRNA2 | CGCTCTTCTAACTCTCATTTTTTCTTCCTGATCGGTGTTTCGTCCTTTCCAC |
| Donor-FOXP1^T176G^-F1 | GAGTAGTTAAAAGCTTCTGC |
| Donor-FOXP1^T176G^-R1 | CTGCTGAAAAGCCAACTGCTGGCCAGCCACCTGCTGTTGCTGTAA |
| Donor-FOXP1^T176G^-F2 | TTACAGCAACAGCAGGTGGCTGGCCAGCAGTTGGCTTTTCAGCAG |
| Donor-FOXP1^T176G^-R2 | CATTCTCTTACTCCAAGAGT |
| Hu-p16-qPCR-F: | CTCGTGCTGATGCTACTGAGGA |
| Hu-p16-qPCR-R: | GGTCGGCGCAGTTGGGCTCC |
| Hu-p21-qPCR-F: | AGGTGGACCTGGAGACTCTCAG |
| Hu-p21-qPCR-R: | TCCTCTTGGAGAAGATCAGCCG |
| Hu-p27-qPCR-F: | ATAAGGAAGCGACCTGCAACCG |
| Hu-p27-qPCR-R: | TTCTTGGGCGTCTGCTCCACAG |
| Hu-p53-qPCR-F: | CCTCAGCATCTTATCCGAGTGG |
| Hu-p53-qPCR-R: | TGGATGGTGGTACAGTCAGAGC |
| Hu-RB-qPCR-F: | CAGAAGGTCTGCCAACACCAAC |
| Hu-RB-qPCR-R: | TTGAGCACACGGTCGCTGTTAC |
| Hu-ALP-qPCR-F: | AACATCAGGGACATTGACGTG |
| Hu-ALP-qPCR-R: | GTATCTCGGTTTGAAGCTCTTCC |
| Hu-Col1a1-qPCR-F: | GTGCGATGACGTGATCTGTGA |
| Hu-Col1a1-qPCR-R: | CGGTGGTTTCTTGGTCGGT |
| Hu-Actin-qPCR-F: | CCAGCACAATGAAGATCAAGAT |
| Hu-Actin-qPCR-R: | AGAAAGGGTGTAACGCAACTAA |
| Hu-Foxp1-qPCR-F: | GGGGCAGTATGGACAGTGGATGA |
| Hu-Foxp1-qPCR-R: | TTGAGAGGTGTGCAGTAGGCGTG |
| Hu-Runx2-qPCR-F: | CCCAGTATGAGAGTAGGTGTCC |
| Hu-Runx2-qPCR-R: | GGGTAAGACTGGTCATAGGACC |
| Hu-Pparγ-qPCR-F: | AGCCTGCGAAAGCCTTTTGGTG |
| Hu-Pparγ-qPCR-R: | GGCTTCACATTCAGCAAACCTGG |
| Hu-CEBPα-qPCR-F: | AGGAGGATGAAGCCAAGCAGCT |
| Hu-CEBPα-qPCR-R: | AGTGCGCGATCTGGAACTGCAG |
| Hu-FABP4-qPCR-F: | ACGAGAGGATGATAAACTGGTGG |
| Hu-FABP4-qPCR-R: | GCGAACTTCAGTCCAGGTCAAC |
